# Informal care outside the household by older people and cardiovascular health of caregivers: A Cross-Sectional Analysis of the SHARE European study

**DOI:** 10.1371/journal.pone.0359074

**Published:** 2026-10-09

**Authors:** Raquel Cervigón, Evangelos Kontopantelis

**Affiliations:** 1 Technology for Inclusive Living, Escuela Politécnica de Cuenca, Universidad de Castilla-La Mancha, Cuenca, Spain; 2 Division of Informatics, Imaging and Data Science, Faculty of Biology, Medicine and Health, The University of Manchester, Manchester, United Kingdom; 3 Centre for Research in Health Systems Performance, National University of Singapore, Singapore; Independent Medical Researcher and Writer, UNITED KINGDOM OF GREAT BRITAIN AND NORTHERN IRELAND

## Abstract

Social isolation is an established cardiovascular risk factor, but the health implications of providing social support remain understudied. We examined whether informal care outside the household is associated with any self-reported cardiovascular disease. We conducted a cross-sectional analysis of 62,881 community-dwelling adults aged 50–84 from 28 countries (SHARE Wave 9, 2021–2022), using multivariable logistic regression with country fixed effects, clustered standard errors, E-values for unmeasured confounding, and sequential exclusions to probe reverse causality. The primary model adjusted for confounders only (age, sex, education, marital status, household wealth, and country); models additionally adjusting for potential mediators were treated as secondary. Among 16,178 informal carers (25.7%), the prevalence of any self-reported cardiovascular disease was 8.8% versus 12.8% in non-carers. In the confounder-adjusted model, informal care was associated with 15% lower odds of any self-reported cardiovascular disease (OR=0.85, 95% CI: 0.78–0.93; E-value 1.39 using the square-root odds-ratio-to-risk-ratio conversion appropriate for a common outcome, or 1.64 treating the odds ratio directly as a risk ratio, which overstates robustness). The association persisted after excluding care recipients and those with functional limitations, attenuated towards the null after excluding those in poor self-rated health, and was similar across age groups and sexes. Inference was robust to a pairs-cluster bootstrap and to population-averaged and random-intercept models, and to a weighted sensitivity analysis using SHARE calibrated weights (OR=0.78, 95% CI: 0.63–0.96).. Because the outcome reflects lifetime self-reported diagnoses and the design is cross-sectional, temporality cannot be established and the findings should be interpreted as hypothesis-generating. Informal care was consistently associated with lower prevalence of any self-reported cardiovascular disease and may be a marker of healthier cardiovascular ageing rather than a demonstrated cause of it.

## Introduction

Cardiovascular diseases (CVDs) remain the leading cause of morbidity and mortality globally, accounting for approximately 17.9 million deaths each year [1]. In Europe, CVDs are responsible for more than 40% of deaths among adults aged 65 years and older, constituting a major source of healthcare expenditure, disability, and loss of quality-adjusted life years [2]. Despite substantial advances in the identification and management of established biomedical risk factors, such as hypertension, dyslipidaemia, diabetes, and smoking, the overall burden of cardiovascular disease remains high. This persistence has prompted increasing attention to social and behavioural determinants as potential targets for cardiovascular prevention [3,4].

Social relationships have long been recognised as relevant to cardiovascular health. Early evidence demonstrated that social integration and social network characteristics predict mortality independently of traditional cardiovascular risk factors [5]. More recent meta-analyses have shown that social isolation and loneliness are associated with increased risks of cardiovascular morbidity and mortality, with effect sizes comparable to those of smoking and physical inactivity [6–8]. In line with this evidence, the American Heart Association has identified social isolation as a modifiable risk factor for cardiovascular disease, highlighting the relevance of social determinants within preventive cardiology [9].

However, most research in this area has focused on structural aspects of social relationships or on the receipt of social support. Far less attention has been paid to the provision of support and assistance to others, despite the possibility that caring behaviours may influence cardiovascular risk through distinct behavioural, psychological, and physiological pathways [10]. From a preventive perspective, this distinction is important, as providing care may represent an active, potentially health-promoting behaviour rather than a marker of vulnerability. Several mechanisms may plausibly link caring behaviours to cardiovascular health. Informal care often involves light-to-moderate physical activity, which is protective against cardiovascular disease [11]. Providing care may also enhance social integration and access to health-related resources [12], improve psychological wellbeing and sense of purpose [13,14], and reduce stress-related physiological dysregulation, including activation of the hypothalamic–pituitary–adrenal axis and sympathetic nervous system [15–17]. Together, these pathways suggest that caring for others could be relevant for cardiovascular prevention, particularly in older populations.

Theoretical frameworks from social gerontology suggest that engagement in socially productive roles may support health in later life. Activity theory and role theory propose that maintaining meaningful social roles promotes wellbeing and buffers against the adverse health effects of social disengagement and role loss [18,19]. The productive ageing framework further emphasises that unpaid activities, including informal care and volunteering, may generate health benefits for older adults who provide them [20,21]. These perspectives align with preventive cardiology’s growing interest in upstream and non-biomedical determinants of cardiovascular risk.

Empirical findings on the cardiovascular health effects of caring behaviours remain mixed. While several studies have reported favourable associations between volunteering or informal care and mortality or cardiovascular outcomes [22–24], these associations are difficult to interpret due to substantial health-based selection. Individuals who provide care are typically healthier and more functionally capable, and reverse causality is a particular concern in aging populations, where declining health constrains participation in socially productive activities [25–27]. Moreover, evidence from caregiving research suggests that the health effects of caring vary by intensity and context, with intensive within-household caregiving often associated with adverse cardiovascular outcomes, in contrast to lower-intensity care provided outside the household [28–31].

In Europe, approximately one quarter of adults aged 50 years and older provide informal assistance to individuals outside their household, including family members, friends, or neighbours [32]. This high prevalence underscores the potential public health relevance of caring behaviours as a modifiable social exposure in later life.

In this study, “informal care outside the household” refers to the provision of practical, non-professional, and unpaid assistance (e.g., household tasks, transport, paperwork, repairs, or gardening) in the previous 12 months to relatives, friends, or neighbours who do not live in the respondent’s household, as captured by the SHARE social-support module. This is distinct from co-residential caregiving, which the literature links to different and potentially adverse health implications.

The present study examines the cross-sectional association between providing informal care to individuals outside the household and any self-reported cardiovascular disease in a large, multinational European sample. We use multiple analytical strategies explicitly designed to probe reverse causality. Because the outcome is derived from a single self-reported item that aggregates heart attack with other heart conditions, we interpret it as any self-reported cardiovascular disease rather than a clinically specific myocardial infarction endpoint, and we frame all findings as associations.

## Materials and methods

### Study design, setting, and period

This was a cross-sectional analysis of Wave 9 (2021–2022) of the Survey of Health, Ageing and Retirement in Europe (SHARE), a harmonised, multinational panel study of community-dwelling adults aged ≥50 years across Europe and Israel [33]. The present analysis used the single most recent wave (Wave 9).

### Study population and data source

SHARE collects data through standardised computer-assisted personal interviews administered by trained interviewers in each participating country, with central harmonisation and quality control by the SHARE Research Data Centre. We analysed the public Wave 9 release (version 9.0.0), accessed on 1 June 2024.

We included community-dwelling respondents aged 50–84 with complete data on the exposure, outcomes, and the covariates used in our models. We excluded nursing-home residents and participants aged 85 years and older, because caring behaviour is uncommon in the latter group and likely reflects advanced frailty and selection effects that would complicate interpretation. The analytical sample comprised 62,881 respondents from 28 countries. We applied complete-case restriction only to the variables used in the present analysis. An earlier derived dataset used for other SHARE-based projects had additionally required complete data on quality-of-life (CASP-12), loneliness, and personality measures; because the present study does not use those variables, conditioning the sample on them would have excluded participants without methodological justification. We therefore derived the sample directly from the full Wave 9 release, restricting only on study-relevant variables. A participant flow diagram is provided (Fig 1). No formal sample-size calculation was performed, as the analysis used all eligible respondents in an existing survey.

**Fig 1:**
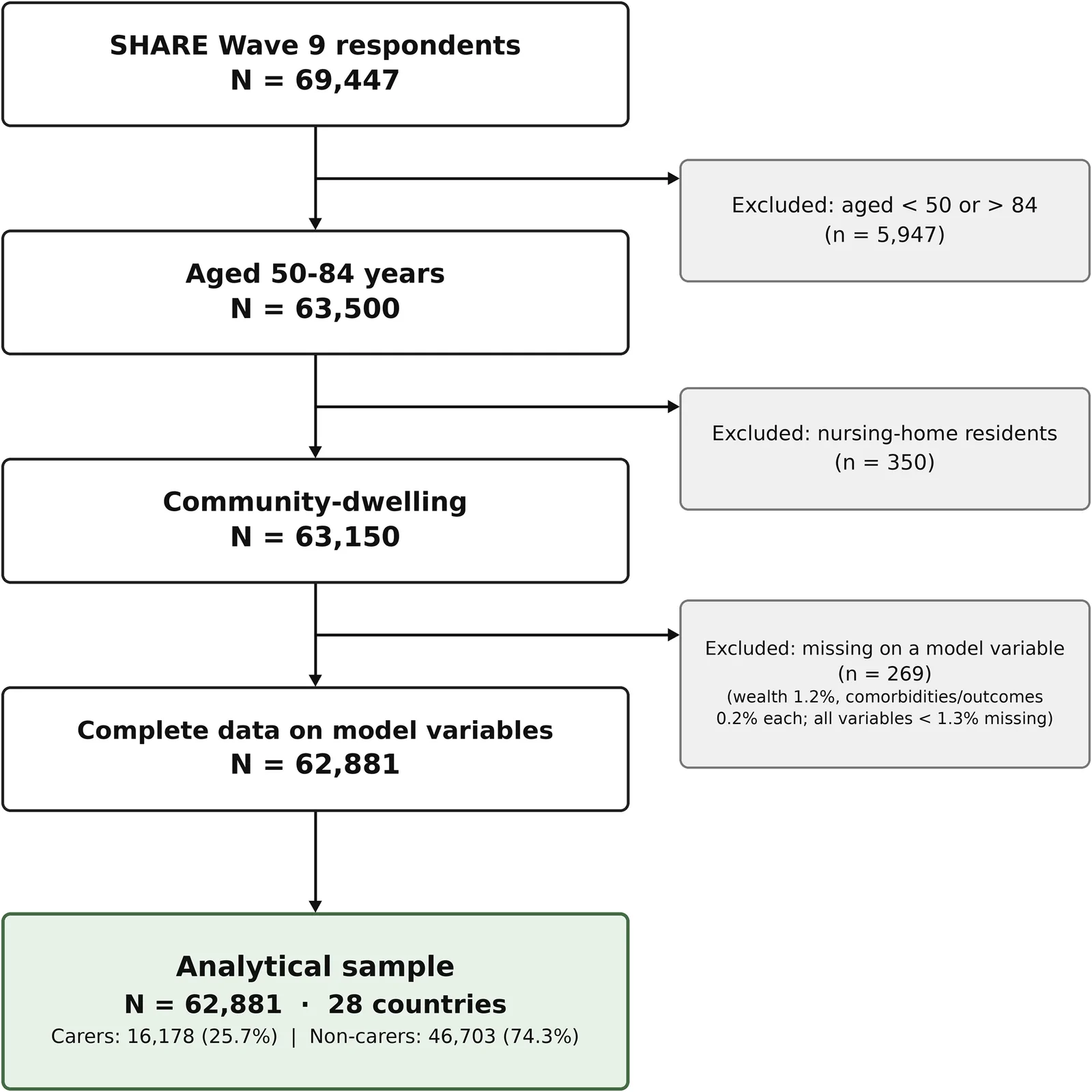
Participant flow diagram. Derivation of the analytical sample (N = 62,881) from the SHARE Wave 9 release, with exclusions at each step. Complete-case restriction was applied only to variables used in the analysis.

### Exposure

The exposure was providing informal care outside the household in the 12 months preceding the interview. Respondents reported whether they had personally given practical assistance—such as help with household tasks, transport, repairs, gardening, or administrative tasks—to family members, friends, or neighbours who did not live with them. The exposure was operationalised as a binary variable (any informal care vs none). Among carers, the number of persons helped was also recorded (0, 1, or ≥2) for threshold analyses. We deliberately excluded within-household caregiving from the exposure, as prior research suggests it has different, potentially adverse, health implications due to chronic stress and physical demands [28].

### Outcomes

The primary outcome was any self-reported cardiovascular disease (heart disease). Respondents were asked whether a doctor had ever told them they had “a heart attack, coronary thrombosis, coronary occlusion, or any other heart problem including congestive heart failure.” We emphasise that this single item aggregates several cardiovascular conditions and is *not* a clinically specific myocardial infarction endpoint; it also reflects lifetime rather than incident disease. We therefore label it any self-reported cardiovascular disease throughout and interpret it accordingly. Secondary outcomes were a combined endpoint (any self-reported cardiovascular disease or stroke) and stroke alone, the latter analysed as exploratory given its greater susceptibility to reverse causality through disabling sequelae.

### Statistical analysis

We used multivariable logistic regression with country fixed effects and standard errors clustered at the country level. Selection of covariates was guided by an explicit consideration of the assumed causal structure, summarised in a directed acyclic graph (Figure 5), distinguishing confounders from variables that plausibly lie on the causal pathway (mediators). The *primary* model estimated the total association, adjusting for confounders only: age (and its square), sex, education (ISCED), marital status, household wealth (signed-log net worth), and country. Two *secondary* models estimated a controlled direct effect by additionally adjusting for potential mediators: Model 2 added hypertension, diabetes, and depression (EURO-D ≥4 [34]); Model 3 further added body mass index, smoking, physical inactivity, and social network size. Because mediator adjustment may introduce overadjustment bias, we present the confounder-only model as primary.

We assessed effect modification across countries by adding a caring ×region interaction across four regional groups (Nordic, Continental, Southern including Israel, and Eastern), grouping Israel with Southern Europe to avoid a category represented by a single country cluster, and comparing models with and without it using a cluster-robust Wald test. A full caring×country interaction would require 27 interaction parameters with only 28 country clusters, resulting in a rank-deficient cluster-robust covariance matrix; the country-level analysis is therefore reported only as a sensitivity analysis in S1 Table. We evaluated model performance using the McFadden pseudo-*R*^2^, the area under the ROC curve (c-statistic), and the Hosmer–Lemeshow test. To assess robustness of inference with a modest number of clusters, we additionally fitted pairs-cluster and wild-cluster bootstraps and, as complementary approaches, a population-averaged generalised estimating equation (exchangeable working correlation) and a logistic model with a random country intercept (S1 Table).

E-values were calculated to assess sensitivity to unmeasured confounding [35]. The E-value represents the minimum strength of association, on the risk-ratio scale, that an unmeasured confounder would need to have with both the exposure and the outcome—conditional on the measured covariates—to fully explain away the observed association. Odds ratios approximate risk ratios only under the rare-outcome assumption; because the outcome was not rare in our sample (prevalence ≈ 12%), that assumption does not hold, and treating the odds ratio directly as a risk ratio would overstate the E-value. We therefore used the square-root transformation (RR ≈ $\sqrt{\text{OR}}$) to obtain an approximate risk ratio before computing the E-value. For transparency, we also report the E- value obtained by treating the odds ratio directly as a risk ratio, which provides an upper (less conservative) bound and is presented only for comparison.

For reverse-causality sensitivity analyses we sequentially excluded markers of poor health, distinguishing *objective* markers (help receipt as a proxy for functional impairment; ADL limitations; depression) from the *subjective* global self-rated health item, since the latter also captures mood and social participation, and conditioning on it—and on a large, disease-enriched subgroup—may induce selection or collider bias. We emphasise that, as a single-wave cross-sectional study, none of these exclusions can establish temporal ordering between caring and disease. Subgroup analyses examined heterogeneity by age (50–64 vs 65–84) and sex, with interaction tests interpreted cautiously given possible limited power. Threshold (“dose”) analyses entered the number of persons helped as indicators, with a linear trend test.

Analyses were not weighted with SHARE calibrated survey weights; instead we accounted for the cross-national structure through country fixed effects and clustering, which is appropriate for estimating within-population associations rather than nationally representative prevalence. A weighted sensitivity analysis using the SHARE calibrated cross-sectional individual weight (cciw_w9) is reported in S1 Table. Complete-case analysis was used: missingness was below 1.3% for every model variable (highest for household wealth, 1.2%), and only 269 of 63,150 eligible respondents (0.43%) were excluded for missing data, so multiple imputation was not employed; per-variable missingness is reported in S1 Table. All analyses were conducted in Stata 17.0 (StataCorp, College Station, TX), with two-sided α=0.05. Reporting follows the STROBE [36] and SAMPL guidelines.

### Ethics

SHARE received ethics approval from the Ethics Council of the Max Planck Society and from ethics committees in participating countries, and all participants provided written informed consent. The data are fully anonymised prior to release; the authors had no access to identifying information.

### Patient and public involvement

Patients and the public were not involved in the design, conduct, or reporting of this secondary analysis of existing survey data.

## Results

### Participant characteristics

Among 62,881 participants, 16,178 (25.7%) provided informal care outside the household (Table 2). The overall prevalence of any self-reported cardiovascular disease was 11.8%. Carers were younger than non-carers (mean age 66.0 vs 69.2 years, standardised mean difference [SMD] = −0.40) and similar in sex distribution (56.0% vs 57.1% female, SMD = −0.02).

Carers had somewhat more favourable cardiovascular risk profiles than non-carers. The prevalence of hypertension was lower among carers (39.9% vs 48.8%, SMD = −0.18), as was diabetes (11.7% vs 16.2%, SMD = −0.13). Depression (EURO-D ≥4) was marginally less common among carers (25.2% vs 26.6%, SMD = −0.03). The prevalence of any self-reported cardiovascular disease was lower among carers (8.8% vs 12.8%, SMD = −0.13), as was stroke (2.5% vs 4.2%, SMD = −0.09).

### Association between informal care and self-reported cardiovascular disease

Informal care was associated with lower odds of any self-reported cardiovascular disease across all model specifications (Table 1, Fig 2). In the primary confounder-only model, carers had 15% lower odds of any self-reported cardiovascular disease (OR=0.85, 95% CI: 0.78–0.93, *p* < 0.001; E-value 1.39 with the square-root odds-ratio-to-risk-ratio conversion appropriate for a common outcome, or 1.64 treating the odds ratio directly as a risk ratio). Adding potential mediators changed the estimate only slightly (Model 2 OR=0.86, 95% CI: 0.78–0.94; Model 3 OR=0.86, 95% CI: 0.79–0.95), indicating little mediation through the measured variables. Associations were similar for the combined endpoint (OR=0.80, 95% CI: 0.74–0.87) and, in exploratory analysis, for stroke (OR=0.70, 95% CI: 0.63–0.79).

**Table 1:** Association Between Informal Care and Cardiovascular Outcomes.

Outcome	Primary (confounders)	Model 2	Model 3	E-value
**Any self-reported CVD — heart disease (primary)**	0.85 (0.78–0.93)	0.86 (0.78–0.94)	0.86 (0.79–0.95)	1.39 / 1.64
Any self-reported CVD (incl. stroke)	0.80 (0.74–0.87)	0.81 (0.74–0.88)	0.83 (0.77–0.91)	1.48 / 1.81
Stroke (exploratory)	0.70 (0.63–0.79)	0.71 (0.64–0.80)	0.80 (0.71–0.90)	1.67 / 2.19

OR (95% CI); standard errors clustered by country. **Primary**: confounder-only total-association model (age, age^2^, sex, education, marital status, household wealth, country). **Model 2**: + hypertension, diabetes, depression (potential mediators). **Model 3**: + BMI, smoking, physical inactivity, social network size (potential mediators). E-values given as (square-root OR-to-RR conversion / OR treated directly as RR) for the primary estimate. The primary outcome derives from a single SHARE item aggregating heart attack and other heart problems; it is not a clinically specific MI endpoint. All *p* < 0.001.

**Fig 2:**
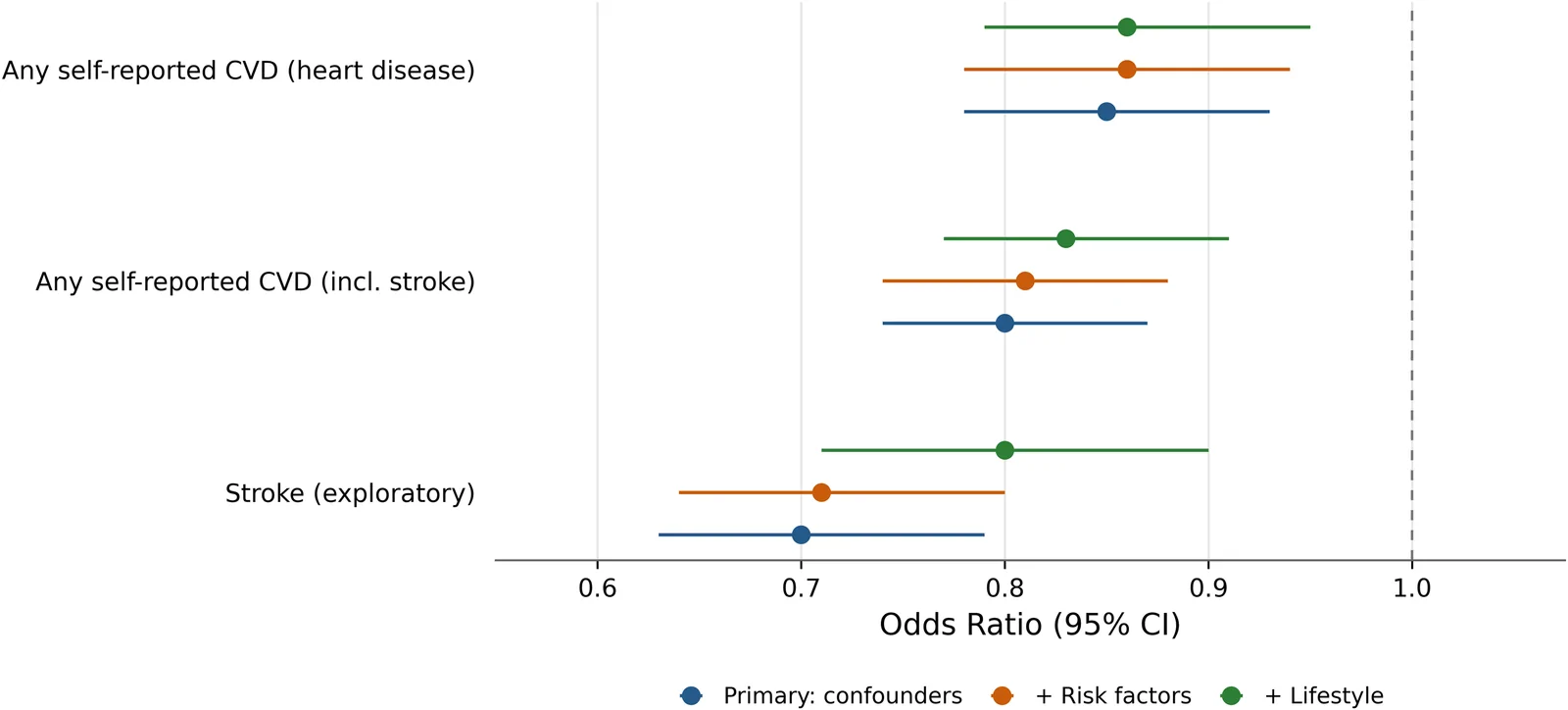
Association between informal care and cardiovascular outcomes. Odds ratios (95% CI) for any self-reported cardiovascular disease (primary outcome), the combined endpoint, and stroke (exploratory). For each outcome, estimates are shown for the primary confounder-only model and the two secondary mediator-adjusted models. Standard errors clustered by country.

The caring×region interaction did not reach conventional statistical significance (cluster-robust Wald $\chi^{2}$=1.56, df = 3, *p* = 0.669). A country-level interaction was examined as a sensitivity analysis, but its cluster-robust covariance matrix was rank-deficient and the result was therefore not used for primary inference (S1 Table). Inference was essentially unchanged under pairs-cluster and wild-cluster bootstraps and under population-averaged (GEE) and random-intercept models (S1 Table), and under the weighted sensitivity analysis using SHARE calibrated cross-sectional weights (OR=0.78, 95% CI: 0.63–0.96; E-value 1.53 / 1.90; S1 Table). For the primary model, the McFadden pseudo-*R*^2^ was 0.06, the c-statistic was 0.69, and the Hosmer–Lemeshow test indicated some lack of fit, as is common in large samples; the modest discrimination is expected given the small set of social predictors.

Fig 3 presents the association by number of persons helped. Compared with non-carers, those caring for one person had lower odds of any self-reported cardiovascular disease (OR=0.83, 95% CI: 0.76–0.91), as did those caring for two or more (OR=0.88, 95% CI: 0.79–0.98). Although a linear-trend test was significant (*p* < 0.001), the two estimates were similar with overlapping confidence intervals, so the data are more consistent with a threshold or participation effect than with a monotonic dose-response gradient (Table 2).

**Table 2:** Baseline Characteristics by Caring Status (N = 62,881).

Characteristic	Non-carers (n = 46,703)	Carers (n = 16,178)	SMD
Age, years, mean (SD)	69.2 (8.4)	66.0 (7.6)	−0.40
Female, %	57.1	56.0	−0.02
Tertiary education, %	21.5	35.1	0.31
Hypertension, %	48.8	39.9	−0.18
Diabetes, %	16.2	11.7	−0.13
Depression (EURO-D ≥4), %	26.6	25.2	−0.03
Any self-reported CVD (heart disease), %	12.8	8.8	−0.13
Stroke, %	4.2	2.5	−0.09

SMD = Standardised Mean Difference.

**Fig 3:**
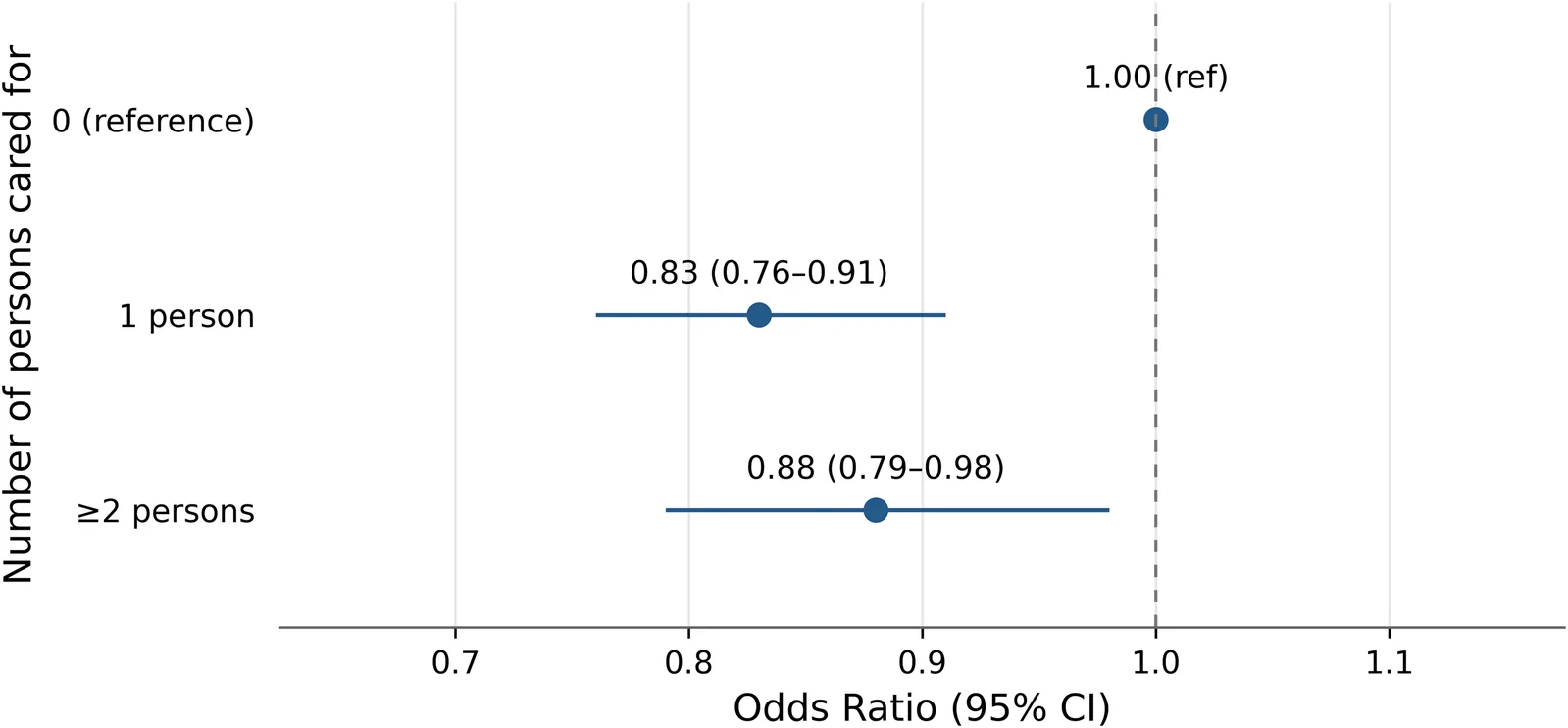
Threshold analysis: number of persons cared for and any self-reported cardiovascular disease. Odds ratios (95% CI) relative to non-carers, for caring for one person or two or more persons. The similar estimates are consistent with a threshold rather than a graded dose-response relationship.

Fig 4 and Table 3 present the association between informal care and cardiovascular risk factors. After adjustment for age, sex, and country, carers had lower odds of hypertension (OR=0.93, 95% CI: 0.88–0.98, *p* = 0.004) and diabetes (OR=0.86, 95% CI: 0.81–0.91, *p* < 0.001). Because these conditions may lie on the causal pathway between caring and cardiovascular health, we present them as descriptive associations rather than as confounders of the primary estimate.

**Table 3:** Association Between Informal Care and Cardiovascular Risk Factors.

Risk Factor	OR (95% CI)	p-value	E-value
Hypertension	0.93 (0.88–0.98)	0.004	1.24 / 1.38
Diabetes	0.86 (0.81–0.91)	<0.001	1.37 / 1.60

Adjusted for age, sex, and country. E-values given as (square-root OR-to-RR conversion / OR treated directly as RR).

**Fig 4:**
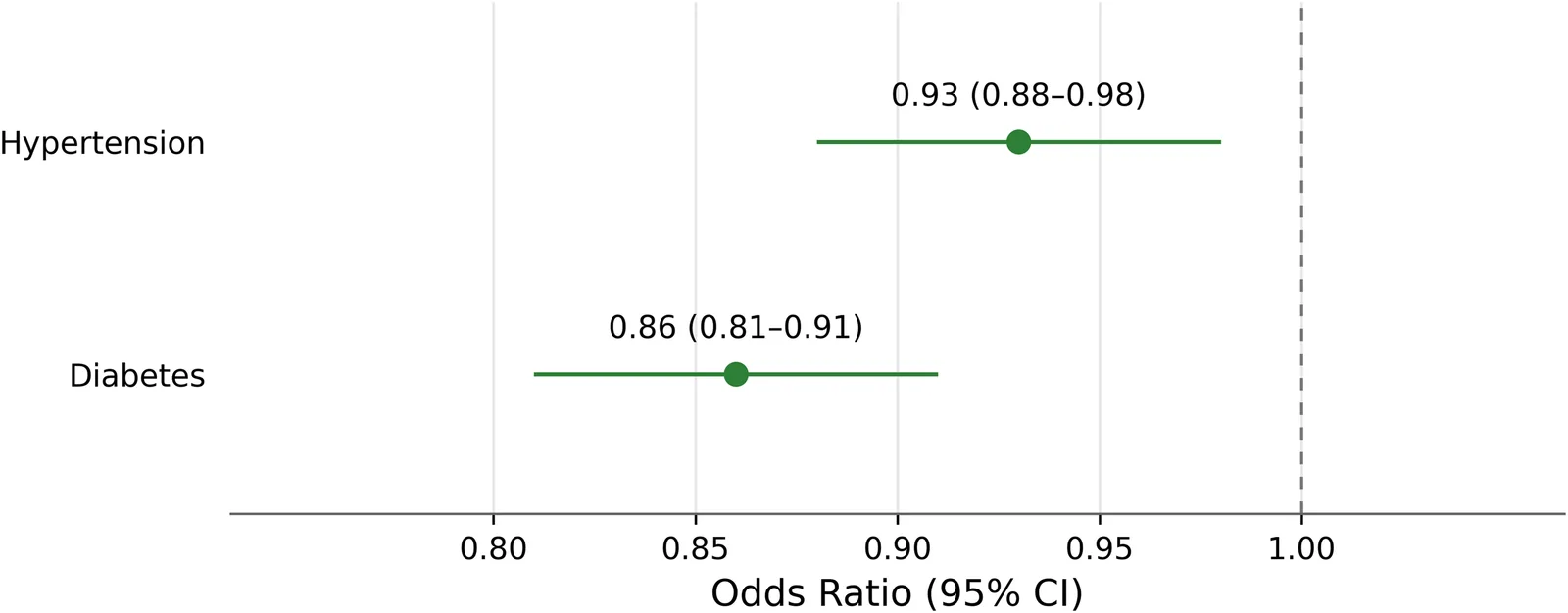
Association between informal care and cardiovascular risk factors. Odds ratios (95% CI) for hypertension and diabetes, adjusted for age, sex, and country. These conditions may lie on the causal pathway and are presented as descriptive associations.

### Sensitivity analyses

Table 4 presents reverse-causality sensitivity analyses for any self-reported cardiovascular disease. The association was robust to excluding *objective* markers of poor health: excluding care recipients (OR=0.81, 95% CI: 0.74–0.89), those with ADL limitations (OR=0.87, 95% CI: 0.80–0.96), or those with depression (OR=0.87, 95% CI: 0.78–0.96). It attenuated towards the null only when excluding those with fair/poor *self-rated* health (OR=0.95, 95% CI: 0.84–1.06). We interpret this cautiously: self-rated health is a broad construct that also reflects mood and social participation, and it excludes a large, disease-enriched fraction of the sample (37%), so conditioning on it may reflect selection or collider bias rather than reverse causality alone. These cross-sectional exclusions cannot establish temporal ordering (Fig 5).

**Table 4:** Sensitivity Analyses for Reverse Causality (any self-reported cardiovascular disease).

Sample	N	OR (95% CI)	Δ%
Full sample	62,881	0.85 (0.78–0.93)	ref
Excluding care recipients	50,835	0.81 (0.74–0.89)	−3.9
Excluding ADL limitations	57,300	0.87 (0.80–0.96)	+3.2
Excluding depression	46,397	0.87 (0.78–0.96)	+3.2
Excluding fair/poor self-rated health	39,712	0.95 (0.84–1.06)	+12.0
Excluding all objective markers	37,436	0.83 (0.74–0.92)	−1.2

OR (95% CI); Δ% = change in OR vs full sample. All rows use the primary confounder-only set (age, age^2^, sex, education, marital status, household wealth, country), identical to Table 1; the full-sample OR therefore matches the primary estimate. The estimate is robust to excluding objective markers of poor health (care receipt, ADL limitations, depression), individually or combined. It attenuates only when excluding fair/poor self-rated health—a broad construct that also reflects mood and social participation and that removes a large, disease-enriched fraction of the sample (37%)—so conditioning on it may reflect selection or collider bias rather than reverse causality alone.

**Fig 5:**
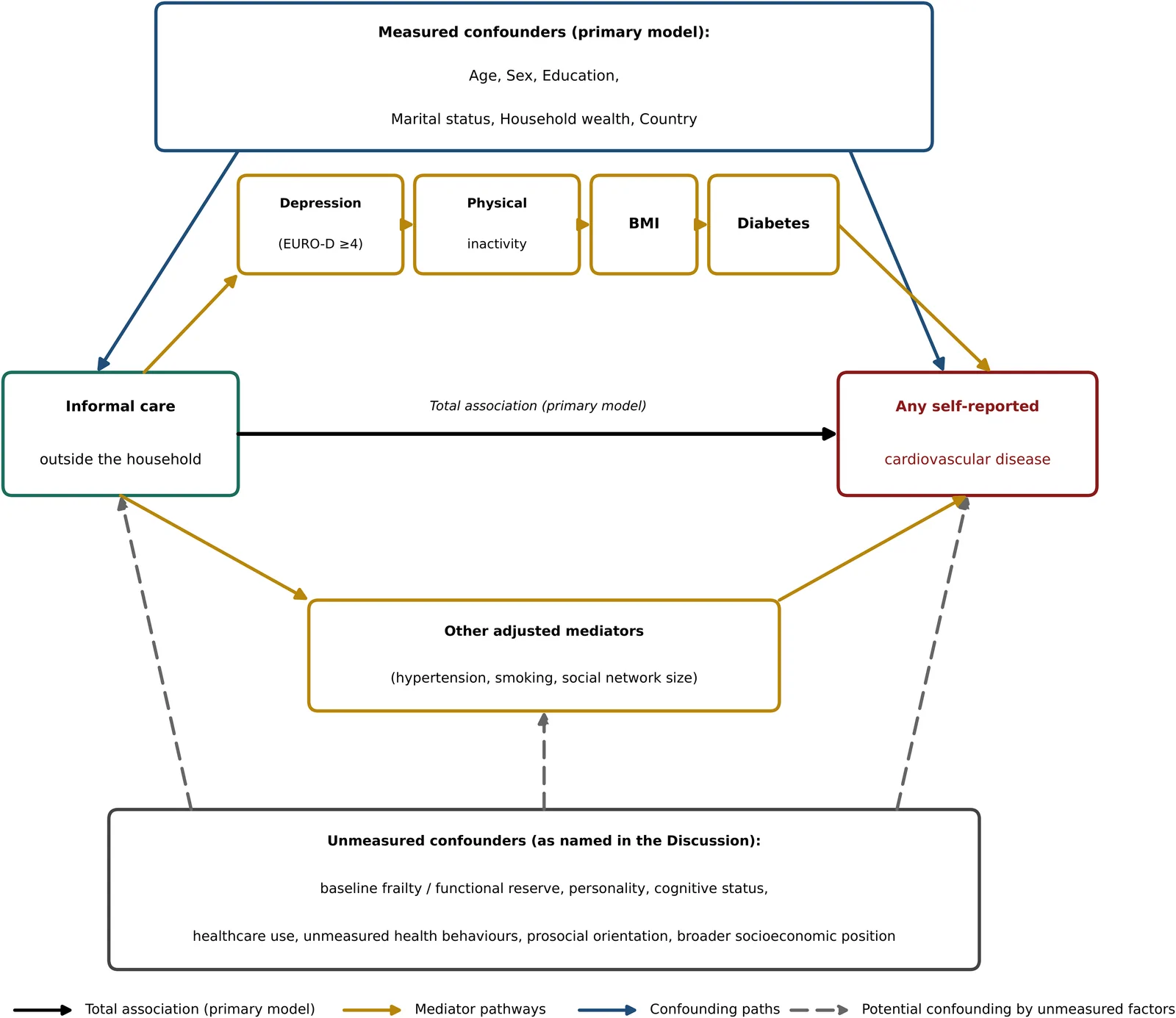
Assumed causal structure (directed acyclic graph). Confounders (adjusted in the primary model) and mediators (adjusted only in secondary models) for the association between informal care and any self-reported cardiovascular disease. Unmeasured confounders (baseline frailty/functional reserve, personality, cognitive status, healthcare use, socioeconomic position) and inter-mediator arrows (e.g., Depression → Physical inactivity → BMI → Diabetes) are depicted.

In the most restrictive analysis, excluding all three groups simultaneously (final n = 33,006), the OR was 0.89 (95% CI: 0.80–1.00). Taken together, these analyses show that the association is not driven solely by the least healthy participants, but they do not exclude reverse causality.

### Subgroup analyses

Table 5 presents subgroup analyses for the primary outcome. The association was similar across age groups: OR=0.88 (95% CI: 0.77–1.01) for ages 50–64 and OR=0.86 (95% CI: 0.78–0.94) for ages 65–84 (p-interaction = 0.72). It was likewise similar by sex: OR=0.87 (95% CI: 0.78–0.96) for women and OR=0.85 (95% CI: 0.75–0.95) for men (p-interaction = 0.30). Because interaction tests may be underpowered, these non-significant results should not be read as establishing the absence of effect modification.

**Table 5:** Subgroup Analyses (Primary Outcome: any self-reported cardiovascular disease).

Subgroup	OR (95% CI)	p-value	p-interaction
**By age group**			0.72
50–64 years	0.88 (0.77–1.01)	0.06	
65–84 years	0.86 (0.78–0.94)	0.002	
**By sex**			0.30
Women	0.87 (0.78–0.96)	0.007	
Men	0.85 (0.75–0.95)	0.005	

Adjusted for age (within strata), diabetes, depression, hypertension, country.

The association with the combined endpoint (any self-reported cardiovascular disease including stroke) was similar to the primary outcome (OR=0.80, 95% CI: 0.74–0.87; E-value 1.48/1.81). The exploratory stroke analysis yielded OR=0.70 (95% CI: 0.63–0.79; E-value 1.67/2.19) in the confounder-only model, attenuating to 0.80 (95% CI: 0.71–0.90) with mediator adjustment; given stroke’s greater susceptibility to reverse causality, we treat this as exploratory.

## Discussion

In this cross-sectional study of 62,881 older adults from 28 countries, providing informal care outside the household was associated with approximately 15% lower odds of any self-reported cardiovascular disease. The association changed little after adjustment for potential mediators, was robust in excluding objective markers of poor health (help receipt, ADL limitations, depression) and was similar between age groups and sexes. It attenuated only when excluding those with fair/poor self-rated health—a broad, partly affective construct on which conditioning, given the large disease-enriched subgroup it removes, may reflect selection or collider bias. Because the design is cross-sectional and the outcome reflects lifetime self-reported diagnoses, we cannot determine temporal ordering, and reverse causality, whereby poorer cardiovascular health reduces the capacity to help—remains a plausible contributor. We therefore interpret the findings as associations rather than evidence of a protective effect.

Our effect size aligns with meta-analytic evidence of 20–25% lower mortality among volunteers [22,23]. Using the square-root odds-ratio-to-risk-ratio conversion appropriate for a common outcome, the E-value for the primary estimate is approximately 1.4 (1.64 if the odds ratio is treated directly as a risk ratio, which overstates robustness). Unmeasured confounding of this magnitude from baseline frailty, functional reserve, personality, cognitive status, healthcare use, or broader socioeconomic position—is entirely plausible in observational social epidemiology, so the E-value does not provide strong reassurance against residual confounding. The distinction between informal care outside the household and intensive within-household caregiving is important: while we found favorable associations for the former, the literature documents adverse cardiovascular effects of the latter [28,29], suggesting that the context and demands of caring behaviour matter.

The similar estimates for caring for one person or two or more are most consistent with a threshold or participation effect rather than a graded dose-response relationship. This pattern is compatible with frameworks emphasising the health correlates of role occupancy and social integration [6,7], but our design cannot identify a mechanism. In the confounder-only model the stroke association appeared stronger than that for any self-reported cardiovascular disease (OR=0.70 vs 0.85), but it attenuated substantially after adjustment for potential mediators (to OR=0.80) and is estimated with less precision. We do not interpret the larger confounder-only estimate as evidence of a greater protective association; rather, stroke is particularly susceptible to reverse causality, because its disabling motor, cognitive, and communication sequelae directly reduce the capacity to help others. We therefore treat stroke as an exploratory outcome throughout. Should any of these associations reflect a genuine effect, plausible pathways include physical activity [11], social integration [12], psychological wellbeing [13,14], and stress buffering [15–17]; these remain hypotheses.

These findings identify informal care as a behavioural correlate of cardiovascular health that merits investigation in prospective cohorts with incident event ascertainment. The dose-response pattern suggests that any level of caring engagement, rather than intensive involvement, may be relevant, although our design cannot identify a mechanism.

## Limitations

Several limitations warrant emphasis. First, and most importantly, the cross-sectional design precludes causal inference. We cannot determine whether caring preceded cardiovascular events, whether cardiovascular events preceded cessation of caring, or whether both are explained by common antecedent factors. The terminology of “association” rather than “effect” is deliberate and essential.

Second, both the exposure (caring) and outcomes (cardiovascular diagnoses) were self-reported. Self-report of cardiovascular diagnoses may be subject to recall error, although studies comparing self-reported diagnoses to medical records suggest reasonable validity [37]. The exposure measure captures whether care was provided but not its intensity, frequency, or duration in detail.

Third, as noted, the E-value (approximately 1.4) indicates that a moderately strong unmeasured confounder could explain our findings. Plausible candidates include personality traits (e.g., conscientiousness, extraversion), unmeasured health behaviours, and aspects of socioeconomic status not captured by education. In particular, unmeasured traits such as prosocial orientation may influence both caring behaviour and cardiovascular risk through shared pathways, including lifestyle choices and stress regulation. Fourth, our cluster-robust standard errors rely on 28 country clusters, which is at the lower bound for reliable inference [38]; for the same reason, effect modification was assessed primarily at the regional level, because the cluster-robust covariance matrix for a full country-level interaction was rank-deficient. We also report pairs-cluster and wild-cluster bootstraps and multilevel models (S1 Table), which were consistent. Interaction tests for subgroup analyses may be underpowered to detect modest effect modification.

Fifth, the prevalence of the self-reported outcome (11.8%) is high, reflecting both the cumulative nature of lifetime self-reported diagnoses and the heterogeneous item that aggregates heart attack with other heart conditions including heart failure—a point we also emphasise in the Methods. Any systematic over-reporting would need to differ by caring status to bias our results. Sixth, we could not examine incident events, which would be more directly relevant to assessing whether caring protects against future cardiovascular disease.

## Conclusions

Informal care outside the household was consistently associated with lower prevalence of any self-reported cardiovascular disease in this large multinational study, with similar estimates across demographic subgroups. Given the cross-sectional design and self-reported lifetime outcome, these findings are best interpreted as indicating that caring may be a marker of healthier cardiovascular ageing or better functional status, rather than a demonstrated cause of it—a hypothesis that warrants testing in longitudinal research with incident events.

## Supporting information

S1 TableRobustness of inference and model diagnostics.Alternative approaches to clustered inference (GEE, pairs-cluster bootstrap, wild-cluster bootstrap, random-intercept), the weighted sensitivity analysis using the SHARE calibrated cross-sectional individual weight (cciw_w9; primary estimate OR=0.78, 95% CI: 0.63–0.96; E-value 1.53 / 1.90), a caring×region interaction test (reported as primary, with the less reliable per-country interaction shown alongside for transparency), model fit statistics, and per-variable missingness.(PDF)

S1 File Analysis code and codebook. Stata do-files and a variable codebook, deposited in a public Figshare repository with a citable DOI (10.6084/m9.figshare.33122441).
(ZIP)
