# Supplementary material for: Informal care outside the household by older people and cardiovascular health of caregivers: A Cross-Sectional Analysis of the SHARE European study
Source: PLoS One. 2026 Oct 9;21(10):e0359074. doi: 10.1371/journal.pone.0359074 (PMC13652321; doi:10.1371/journal.pone.0359074)
Supplement: Supplementary file 1 [file pone.0359074.s001.pdf]

S1 Table. Robustness of inference and model diagnostics

Primary outcome: any self-reported cardiovascular disease (heart disease item). Alternative approaches to clustered inference, cross-country effect modification, model fit, and per-variable missingness, for the association between informal care outside the household and cardiovascular outcomes.

Panel A — Alternative approaches to inference with country structure

Method	OR for caring	95% CI
Primary: logistic + country fixed effects, cluster-robust SE	0.85	0.78–0.93
Population-averaged (GEE, exchangeable working correlation)	0.85	0.80–0.91
Pairs-cluster bootstrap (resampling countries)	0.85	0.78–0.92
Wild-cluster bootstrap (`boottest`, 9999 reps)[a]	0.85	0.78–0.93
Random-intercept (country) logistic model	0.85	0.79–0.91
Weighted (SHARE calibrated weight, cciw_w9)	0.78	0.63–0.96

Alternative inferential approaches yielded broadly similar estimates. The weighted sensitivity analysis was somewhat stronger but its confidence interval overlapped the primary estimate. [a]For the wild-cluster bootstrap, the point estimate and confidence interval shown are those from the primary model; the wild-bootstrap test gave $p = 0.0015$ *(9,999 replications). E-value for the weighted estimate: 1.53 (square-root OR-to-RR approximation) / 1.90 (OR treated directly as RR).*

Panel B — Cross-region effect modification (primary: 4 regions) and country-level sensitivity analysis

Test	χ^2	df	p
Caring × region interaction (4-region grouping; cluster-robust Wald test; primary)	1.56	3	0.669
Sensitivity: 2-block grouping (East+Israel vs. West)	1.24	1	0.265
Caring × country interaction (likelihood-ratio test; sensitivity analysis)	50.09	27	0.0044
Caring × country interaction (Wald test, non-cluster-robust; sensitivity analysis)	44.26	27	0.0194

Geographic effect modification was assessed using four regional groupings (Nordic, Continental, Southern+Israel, and Eastern), with Israel grouped with Southern Europe to avoid defining a regional category represented by a single country cluster. There was no evidence of effect modification by region in this primary specification (χ^2(3)=1.56, $p = 0.669$*). Results were similar in a coarser two-block grouping (East+Israel vs. West; χ^2(1)=1.24,* $p = 0.265$*). A full caring×country interaction requires 27 additional parameters with only 28 country clusters; its cluster-robust covariance matrix was rank-deficient, so the ordinary likelihood-ratio and non-cluster-robust Wald tests are shown only as sensitivity analyses and should be interpreted cautiously.*

Panel C — Model fit and discrimination (primary model)

Diagnostic	Value
McFadden pseudo-R^2	0.0625
Area under ROC curve (c-statistic)	0.6862
Hosmer–Lemeshow goodness-of-fit	χ^2(8)=28.99, $p = 0.0003$

The modest discrimination is expected given the limited predictor set. The Hosmer–Lemeshow test is highly sensitive in large samples and should be interpreted alongside the other diagnostics rather than in isolation.

Panel D — Per-variable missingness (model variables; base: age 50–84, community-dwelling, N = 63,150)

**Variable**	n **missing**	%
Exposure: care given outside household (ghto)	0	0.00
Outcome: any self-reported CVD (ph006d1)	149	0.24
Outcome: stroke, exploratory (ph006d4)	149	0.24
Sex	0	0.00
Education (ISCED)	0	0.00
Marital status	0	0.00
Hypertension	149	0.24
Diabetes	149	0.24
Depression (EURO-D)	0	0.00
Body mass index	0	0.00
Smoking	0	0.00
Physical inactivity	0	0.00
Social network size	153	0.24
Household wealth	781	1.24

Total excluded for missingness on any model variable: 269 of 63,150 (0.43%). Missingness was below 1.3% for every model variable; given this very low level and the cross-sectional design, complete-case analysis was used and multiple imputation was not employed. Missingness is computed after the age and community-dwelling restrictions, i.e., on the set eligible for the model.
