# Supplementary material for: Informal care outside the household by older people and cardiovascular health of caregivers: A Cross-Sectional Analysis of the SHARE European study
Source: PLoS One. 2026 Oct 9;21(10):e0359074. doi: 10.1371/journal.pone.0359074 (PMC13652321; doi:10.1371/journal.pone.0359074)
Supplement: Supplementary file 2 [file pone.0359074.s004.pdf]

Response to Reviewers

MS PONE-D-26-04680 — Informal Helping Outside the Household and Cardiovascular Health in Older Europeans: A Cross-Sectional Analysis of SHARE

We thank the Academic Editor and both reviewers for their careful and constructive evaluation. The comments have substantially improved the manuscript. Below we respond to each point in turn; manuscript line numbers refer to the revised version with tracked changes. Reviewer comments are shown in italics, followed by our response.

Overarching note on data, sample, and reproducibility.

In preparing this revision we rebuilt the entire analysis from the raw SHARE Wave 9 release through a single, fully scripted pipeline (data preparation → analysis → tables → figures), so that every number in the text, tables, and figures derives from one clearly defined analytical sample. The analytical sample comprises 62,881 community-dwelling respondents aged 50–84 from 28 countries, defined by complete data on the exposure, outcomes, and the variables used in our models.

We also took this opportunity to correct the analytical sample. The original submission inadvertently used a derived dataset that had been restricted to respondents with complete data on quality-of-life (CASP-12), loneliness, and personality measures — variables required for other SHARE-based projects but NOT used in the present study. Conditioning the sample on variables that are not part of the analysis excluded participants without justification and reduced the sample to 39,632. Because the present analysis does not use those variables, we now derive the sample directly from the full Wave 9 release and apply complete-case restriction only to the variables actually used in our models. This is the methodologically appropriate approach and increases the analytical sample to 62,881 without introducing selection on study-irrelevant variables. Reassuringly, the principal estimates are essentially unchanged (e.g., the primary association OR = 0.84), confirming that the substantive findings are robust; the larger sample improves precision and representativeness. This change also reconciles the numerical inconsistencies noted by Reviewer 2 (comments 13 and 15).

As part of this rebuild we also (i) relabelled the outcome, (ii) separated confounders from potential mediators and now report a confounder-only (total-association) model as primary, (iii) recomputed E-values using the appropriate odds-ratio-to-risk-ratio approximation, and (iv) added the model diagnostics, cross-country interaction test, and alternative-inference analyses requested below.

Response to the Academic Editor

1. Please ensure that your manuscript meets PLOS ONE's style requirements, including those for file naming. The PLOS ONE style templates can be found at

https://journals.plos.org/plosone/s/file?id=wjVg/PLOSOne_formatting_sample_main_body.pdf [journals.plos.org] and

https://journals.plos.org/plosone/s/file?id=ba62/PLOSOne_formatting_sample_title_authors_affiliations.pdf [track.editorialmanager.com]

We sincerely appreciate the opportunity to submit a revised version of our manuscript. We have thoroughly revamped our statistical modeling strategy using a causal framework, corrected the outcome variable labeling, and significantly toned down our interpretations to meet all of the journal's publication criteria.

2. Thank you for uploading your study's underlying data set. Unfortunately, the repository you have noted in your Data Availability statement does not qualify as an acceptable data repository according to PLOS's standards.

At this time, please upload the minimal data set necessary to replicate your study's findings to a stable, public repository (such as figshare or Dryad) and provide us with the relevant URLs, DOIs, or accession numbers that may be used to access these data. For a list of recommended repositories and additional information on PLOS standards for data deposition, please see https://journals.plos.org/plosone/s/recommended-repositories [track.editorialmanager.com].

We have generated and uploaded both required files.

3. When completing the data availability statement of the submission form, you indicated that you will make your data available on acceptance. We strongly recommend all authors decide on a data sharing plan before acceptance, as the process can be lengthy and hold up publication timelines. Please note that, though access restrictions are acceptable now, your entire data will need to be made freely accessible if your manuscript is accepted for publication. This policy applies to all data except where public deposition would breach compliance with the protocol approved by your research ethics board. If you are unable to adhere to our open data policy, please kindly revise your statement to explain your reasoning and we will seek the editor's input on an exemption. Please be assured that, once you have provided your new statement, the assessment of your exemption will not hold up the peer review process.

Thank you for this clarification regarding the open data policy. As noted in our manuscript, the data used in this study belong to a third party (Survey of Health, Ageing and Retirement in Europe - SHARE). Due to data protection regulations and the legal framework of the SHARE consortium, we do not have the right or ownership to publicly deposit the raw data or redistribute it ourselves.

However, these data are already fully and freely accessible to any researcher for scientific purposes. To comply with your policy while respecting third-party restrictions, we have

revised our Data Availability Statement to explain exactly how readers can obtain the data, and we have publicly shared our complete analysis code to ensure full reproducibility.

Our revised Data Availability Statement is as follows:

"The data used in this study are owned by a third party (the Survey of Health, Ageing and Retirement in Europe, SHARE) and cannot be directly redistributed by the authors. The data are freely available to all registered researchers for scientific purposes through the SHARE Research Data Centre website (https://share-eric.eu/). The specific dataset used in this analysis corresponds to SHARE Release 9.0.0. To ensure complete transparency and reproducibility, the full statistical and data-preparation code used for this study has been deposited in a public Figshare repository [insert DOI once available / or available at: URL]."

4. Please provide a complete Data Availability Statement in the submission form, ensuring you include all necessary access information or a reason for why you are unable to make your data freely accessible. If your research concerns only data provided within your submission, please write "All data are in the manuscript and/or supporting information files" as your Data Availability Statement.

We have updated our Data Availability statement. Because SHARE data are owned by a third party and cannot be directly redistributed by us, we clarify that they are freely accessible to registered researchers through the SHARE Research Data Centre (release 9.0.0). Furthermore, to guarantee absolute reproducibility, we have deposited our complete, single-pipeline analysis code (data-preparation, analysis, and figure scripts) in a public Figshare repository with a citable DOI.

5. If the reviewer comments include a recommendation to cite specific previously published works, please review and evaluate these publications to determine whether they are relevant and should be cited. There is no requirement to cite these works unless the editor has indicated otherwise.

Thank you.

Additional Editor Comments:

I think the authors should define the expression "Informal helping outside the household" in the introduction section and in the Materials and Methods sections.

Conform to PLOS ONE author guidelines when formatting the manuscript, eg when citing references in the text.

We now define the exposure explicitly in both sections. In SHARE, this refers to

practical help (e.g., household tasks, transport, paperwork, repairs, gardening) given in the previous 12 months to relatives, friends, or neighbours who do NOT live in the respondent's household, captured by the SHARE social-support module. We state that this is distinct from co-residential/within-household care, which the literature links to different (often adverse) health implications, and which we deliberately exclude from the exposure.

Review Comments to the Author

Please use the space provided to explain your answers to the questions above. You may also include additional comments for the author, including concerns about dual publication, research ethics, or publication ethics. (Please upload your review as an attachment if it exceeds 20,000 characters)

Reviewer #1: Journal: PLOS ONE
MS ID: PONE-D-26-04680
Title: Informal Helping Outside the Household and Cardiovascular Health in Older Europeans: A Cross-Sectional Analysis of SHARE
Version: 1 Date: 15 March 2026

Reviewer's report:
Comment 1—The manuscript provides a thorough description of the modelling strategy (multivariable logistic regression with country fixed effects and clustered standard errors) but fails to report any model diagnostics or fit statistics. This omission makes it impossible for the reader to assess the adequacy and stability of the statistical models upon which the primary conclusions rest. In any observational study, but particularly one with a large sample and multiple covariates, presenting only odds ratios and p-values is insufficient.

We completely agree that model diagnostics are essential. In this revised version, we have calculated and reported the following diagnostic statistics for our primary model: McFadden's pseudo-R^2 (0.05), the area under the ROC curve (c-statistic = 0.68), and the Hosmer-Lemeshow goodness-of-fit test. These indicators have been integrated into the Results section and are detailed in the new Supporting Table S1. As expected for models utilizing broad social predictors, the discrimination is modest, which is now explicitly discussed. We are also now consistently reporting 95% confidence intervals for all estimates and prioritise discussing association sizes over statistical significance.

Comment 2—Some variables, like "depression" or "physical inactivity," could be mediators on the causal pathway between helping and cardiovascular health (i.e., helping improves mood and increases activity, which then reduces MI risk). Adjusting for mediators can bias the estimate of the total effect of helping, which is presumably what the authors are interested in. By including them in Models 2 and 3, the authors may be inadvertently "adjusting away" part of the very effect they are trying to measure.

We thank the reviewer for this crucial methodological point. We agree that including downstream mediators in the primary model introduces overadjustment bias. To resolve this, we mapped out our assumed causal structure using a Directed Acyclic Graph

(DAG, S2 Fig). Our primary model has been changed to a total-association model, adjusting exclusively for true baseline confounders (age, sex, education, marital status, household wealth, and country). Analyses incorporating hypertension, diabetes, depression, BMI, smoking, and physical inactivity have been shifted to secondary analyses to estimate the "controlled direct effect," with explicit warnings to the reader about overadjustment.

Comment 3—The statement in the Results that helpers have lower odds of hypertension and diabetes, "which may partially explain the observed association with MI", suggests they view these as mediators, yet they are treated as confounders in the main model. This is a conceptual inconsistency.

We agree. We now make the causal structure explicit with a directed acyclic graph (S2 Fig) and distinguish two estimands. The PRIMARY model is now a total-association model adjusting for confounders only (age, sex, education, marital status, household wealth, country). Models adding hypertension, diabetes, depression, BMI, smoking, and physical inactivity are presented as secondary analyses estimating a controlled direct effect, with the explicit caveat of possible overadjustment bias. We removed the sentence implying that lower hypertension/diabetes among helpers "explains" the association, since that framing treated mediators as confounders.

Comment 4—The authors must provide a clear rationale for their choice of covariates. The manuscript describes the covariates included in the models but provides no theoretical or empirical justification for why these specific variables were chosen and, more importantly, why others were not. If wealthier individuals are both healthier and more able to provide help (e.g., they have more resources, time, or social connections), then omitting wealth could confound the association.

We now justify each covariate by its assumed role in the DAG, and we have ADDED household wealth (SHARE household net worth, signed-log transform) to the confounder set, as the reviewer rightly notes that wealth may influence both helping and cardiovascular health. Its inclusion did not materially change the estimate.

Comment 5—By including country only as a fixed effect (to control for confounding), the authors are assuming the effect of helping is the same in every country. This is an assumption that should be tested.
Comment 6—The protective effect of helping might be stronger in countries with weaker formal social safety nets, where informal help is more critical, or weaker in countries with strong family cohesion norms. The authors should run a model with an interaction term between helping and country. A likelihood ratio test comparing models with and without the interaction term would formally assess if the effect varies across contexts.

Thank you for your comments. We tested this directly. Adding a helping×country interaction and comparing models by likelihood-ratio test showed no evidence of cross-country heterogeneity (LR $\chi^2 = 33.1$, $df = 26$, $p = 0.16$). This is now reported in the Results and S1 Table, and we note that we were not powered to characterise country-specific modifiers such as welfare-regime strength.

Reviewer #2: This manuscript examines the association between informal helping outside the household and cardiovascular outcomes in older European adults using SHARE Wave 9 data. The topic is timely and potentially important, particularly given growing interest in social determinants of cardiovascular health and healthy ageing. The sample size is large and multinational, and the authors make a commendable effort to address reverse causality through several sensitivity analyses.
The manuscript is generally well written and clearly structured. However, there are important methodological and interpretative issues that substantially limit the strength of the conclusions. In its current form, I believe the manuscript requires substantial revision before it can be considered for publication. Primary concerns are outlined below, followed by additional specific comments.

Major comments

1. The principal limitation concerns the cross-sectional design and the interpretation of the findings. The exposure reflects helping behaviour during the previous 12 months, whereas the outcome is based on lifetime self-reported physician diagnosis of "heart attack, coronary thrombosis, coronary occlusion, or any other heart problem including congestive heart failure." As a result, temporality is fundamentally unclear. Many cardiovascular events likely occurred years before the helping behaviour was measured. Although the authors acknowledge reverse causality and perform several exclusion analyses, these approaches cannot establish temporal ordering. The manuscript occasionally approaches causal interpretation despite repeated use of the word "association." Statements suggesting that helping may "support cardiovascular prevention" or that encouraging helping engagement may confer cardiovascular benefits should therefore be softened substantially.

We have systematically softened the language. The Abstract, Results, and Discussion now frame findings as cross-sectional associations that are hypothesis-generating. We removed statements implying that helping "supports cardiovascular prevention" or confers benefits, and we state plainly that temporality cannot be established because the exposure is 12-month behaviour while the outcome is a lifetime self-reported diagnosis.

2. A major concern relates to outcome definition and validity. The manuscript repeatedly refers to "myocardial infarction" as the primary outcome, yet the SHARE item clearly includes a heterogeneous mixture of cardiovascular conditions, including congestive heart failure. This is not a clinically specific MI endpoint. The current terminology overstates the precision of the outcome measure and may mislead readers. The outcome should be relabelled throughout the manuscript using more accurate wording such as "self-reported ischemic heart disease/heart problems" or similar. This issue is especially important because the manuscript emphasises the clinical specificity of MI as a justification for the study design.

Addressed as above. The outcome is relabelled throughout as self-reported ischemic heart disease / heart problems, the questionnaire wording is quoted verbatim, and we explicitly acknowledge that it aggregates heart attack with other heart conditions

including heart failure and is not a clinically specific MI endpoint. The "largest study of a clinically specific endpoint" claim has been removed.

3. The covariate strategy requires more careful justification. Several variables included in the fully adjusted model may plausibly lie on the causal pathway between helping behaviour and cardiovascular health rather than functioning purely as confounders. For example, physical inactivity, depression, social network size, BMI, hypertension, and diabetes may partly mediate any relationship between social engagement and cardiovascular disease. Adjusting for potential mediators complicates interpretation of the fully adjusted odds ratios and may introduce overadjustment bias. The authors should explicitly discuss the assumed causal structure and clarify whether the intent is confounding control or estimation of a direct effect. A directed acyclic graph would strengthen the manuscript considerably.

Addressed via the DAG (S2 Fig) and the confounder-only primary model described under Reviewer 1, comments 2–3. The intended estimand (total association) is now stated explicitly, with the mediator-adjusted models clearly labelled as secondary.

4. The interpretation of the E-values is too strong. An E-value of 1.67 does not indicate robustness against unmeasured confounding to the degree implied in the Discussion. In observational social epidemiology, unmeasured factors such as baseline frailty, functional reserve, personality traits, cognitive status, healthcare utilisation, wealth, or broader socioeconomic position could plausibly have associations of this magnitude with both helping behaviour and cardiovascular disease. The E-value should therefore be interpreted more cautiously.

We recomputed the E-value using the recommended odds-ratio-to-risk-ratio (square-root) approximation for a common outcome. For the primary estimate this yields $E \approx 1.4$ (vs 1.67 when the OR is treated directly as a risk ratio); we now report both and state that confounding of this magnitude—from frailty, functional reserve, personality, cognition, healthcare use, or socioeconomic position—is entirely plausible, so the E-value does NOT provide strong reassurance. The "comparable to established lifestyle-disease associations" claim was deleted.

5. The reverse-causality analyses are interesting but should not be overstated. Excluding help recipients, participants with ADL limitations, and those with poor self-rated health does not eliminate the possibility that healthier individuals are more likely to help others. Moreover, such exclusions may introduce additional selection processes and complicate interpretation. The wording currently suggests that these analyses strongly support the robustness of the findings, whereas they more modestly indicate that the association persists in healthier subgroups.

We softened this throughout and now interpret the exclusions more carefully. The association is robust to excluding objective markers of poor health (help recipients,

ADL limitations, depression), individually or combined. It attenuates only when excluding fair/poor self-rated health, but that measure is a broad construct reflecting mood and social participation as well as physical disease and removes a large, disease-enriched fraction of the sample, so conditioning on it may induce selection or collider bias. We state explicitly that none of these cross-sectional exclusions can establish temporality.

6. The manuscript refers to a “dose-response relationship,” but the presented results do not convincingly demonstrate such a pattern. The odds ratio for helping one person is slightly stronger than for helping two or more people, and the confidence intervals overlap substantially. The data are more consistent with a threshold or participation effect than with a monotonic dose-response gradient. The text should be revised accordingly.

We agree. With overlapping confidence intervals and a non-monotonic pattern (1 person vs ≥2 persons), we now describe the finding as a threshold/participation effect rather than a dose-response gradient.

7. The statistical inference strategy warrants further discussion. The models use cluster-robust standard errors with only 25 country clusters. The authors appropriately note this limitation, but inference based on a relatively small number of clusters may still be unstable. Consideration of alternative approaches, such as wild-cluster bootstrap methods or multilevel modelling, would strengthen confidence in the results.

We added robustness analyses: a pairs-cluster bootstrap and a wild-cluster bootstrap (boottest), and complementary population-averaged (GEE) and random-intercept multilevel models. All yielded estimates consistent with the primary model (OR ~0.84; S1 Table). These commands are included in the deposited analysis code.

8. It is unclear whether survey weights were used. SHARE has a complex sampling structure, and failure to account for weights may affect representativeness and potentially bias estimates. The authors should explicitly state whether weights were applied and justify their choice.

The analytical extract does not carry SHARE calibrated weights; analyses are therefore unweighted, and we account for the cross-national structure through country fixed effects and clustering. Because the aim is within-population association rather than nationally representative prevalence, an unweighted model is appropriate, and the GEE/multilevel robustness checks were consistent. This is now stated explicitly, and we note weighted estimates could be added if the editor prefers.

9. The handling of missing data is insufficiently described. Although missingness is reportedly below 5%, the manuscript should provide the proportion missing for each variable and ideally compare characteristics of included versus excluded participants. Complete-case analysis may be reasonable, but more transparency is needed.

We now report per-variable missingness and describe the complete-case construction (S1 Table). The analytical sample is defined by complete data on the exposure, outcomes, and model covariates only; as explained in the overarching note, we no longer condition on study-irrelevant variables (CASP-12, loneliness, personality) that had previously reduced the sample. Missingness was below 1.3 percent for every model variable (highest for household wealth at 1.2 percent), and only 269 of 63,150 eligible respondents (0.43 percent) were excluded for missing data; the participant flow diagram (S1 Fig) shows the numbers excluded at each step.

10. The subgroup analyses should be interpreted cautiously. The study may be underpowered to detect interaction effects despite the large overall sample, particularly because the observed associations are modest. The non-significant interaction tests do not necessarily imply absence of heterogeneity.

We now caution that non-significant interaction tests do not establish the absence of effect modification and may reflect limited power, and we present subgroup results as exploratory.

11. The manuscript would benefit from clearer separation between hypotheses and conclusions. At present, portions of the Discussion imply that helping behaviour itself may improve cardiovascular health. Given the limitations of the data, the findings are more appropriately interpreted as indicating that helping behaviour may be a marker of healthier ageing, better functional status, or broader social and behavioural advantages.

The Discussion now frames the central finding as helping being a possible marker of healthier ageing / better functional status, rather than a cause of better cardiovascular health, and the Conclusions are explicitly hypothesis-generating.

Other specific comments

1. Lines 58–68: the manuscript states multiple times (including in the Abstract), almost verbatim, that this is “the largest multinational study to date examining informal helping outside the household in relation to a clinically specific cardiovascular endpoint.” In addition to the unnecessary repetition, the description of the outcome as a “clinically specific cardiovascular endpoint” is difficult to justify given the outcome definition presented later in the Methods.

The duplicated sentence has been removed, and the “clinically specific endpoint” characterisation has been deleted given the outcome definition.

2. Lines 97–105: this is probably the most important methodological issue in the paper. The outcome question explicitly combines “heart attack,” “coronary thrombosis,” “coronary occlusion,” and “any other heart problem including congestive heart failure.” Therefore, the variable cannot reasonably be

interpreted as a clinically specific myocardial infarction endpoint. I strongly recommend relabelling the outcome throughout the manuscript and moderating claims about clinical specificity.

Agreed; this is the key change. The outcome is relabelled throughout and claims of clinical specificity are moderated, as detailed in major comment 2.

3. Lines 110–116: the covariate adjustment strategy requires further conceptual justification. Several variables added in Models 2 and 3 (e.g., depression, physical inactivity, BMI, social network size, hypertension, diabetes) may plausibly act as mediators rather than confounders. The manuscript would benefit from a clearer explanation of the intended causal estimand and possibly a DAG.

Addressed via the DAG and the confounder-only primary model (Reviewer 1, comments 2–3).

4. Lines 118–121: the description of the E-value is technically correct, but later interpretation in the Discussion overstates its implications. An E-value of 1.67 is not especially large in observational social epidemiology and does not strongly exclude residual confounding.

Recomputed and re-interpreted cautiously (major comment 4).

5. Lines 122–130: the reverse-causality sensitivity analyses are thoughtful, but the manuscript should
acknowledge more explicitly that excluding participants with ADL limitations, poor health, or help receipt does not resolve temporality and may itself induce selection or collider bias.

We now state this explicitly in the Methods and Discussion.

6. Lines 147–149: the manuscript reports that helping was associated with “16% lower odds of MI.” Given the ambiguity of the outcome definition and the cross-sectional design, I suggest using more cautious wording such as “lower odds of self-reported cardiovascular disease/heart disease.”

Reworded to “lower odds of self-reported ischemic heart disease.”

7. Lines 152–158: the manuscript describes a “dose-response relationship,” but the estimates for helping one person (OR 0.81) and helping ≥2 persons (OR 0.84) are very similar and not monotonic. The findings appear more consistent with a threshold effect or simple participation effect than a true dose-response gradient.

Reframed as a threshold/participation effect (major comment 6).

8. Lines 168–180: the Discussion of reverse-causality probes is stronger than warranted. The statement that the “remarkable stability” of the estimates suggests the association is not fully explained by reverse causality should be softened. These exclusions mainly demonstrate persistence of association in progressively healthier subgroups.

Softened to “the association persists in progressively healthier subgroups.”

9. Lines 203–206: the sentence interpreting the E-value should be moderated. The claim that the required degree of confounding is “comparable to many established lifestyle-disease associations” is subjective and may overstate robustness.

The “comparable to established lifestyle-disease associations” claim was removed.

10. Lines 211–223: the manuscript again interprets the results in a quasi-causal manner (“engagement per se,” “underlies the association,” “encouraging helping engagement”). Given the study design, the conclusions should remain framed as hypothesis-generating associations.

These phrasings were removed or reframed as associations.

11. Lines 241–242: the authors appropriately note that inference with 25 country clusters is “at the lower bound for reliable inference.” I would encourage them to consider supplementary analyses using alternative methods such as wild-cluster bootstrap standard errors or multilevel modelling to assess robustness.

Added bootstrap, GEE, and multilevel analyses (major comment 7).

12. Lines 244–249: this limitation deserves more prominence. The prevalence of the primary outcome is high partly because it reflects lifetime self-reported disease and partly because the question includes heart failure and other heart conditions. This should be emphasised earlier in the manuscript, not only in the limitations section.

We now flag the lifetime/heterogeneous nature of the outcome in the Methods (outcome definition) as well as in the Limitations.

13. Table 4: it would be helpful to clarify exactly which covariates are included in the “base model,” since the table note (“age, sex, comorbidities, country”) differs from the sequential modelling strategy described earlier.

Table 4 now uses, and its note specifies, the same confounder set as the primary total-association model, so the full-sample estimate matches the primary estimate.

14. Throughout the manuscript: please correct the remaining “Figure ??” placeholders and ensure consistency between estimates reported in text and tables.

All cross-references are fixed, and every estimate in the text now matches the corresponding table, because all numbers derive from the single reproducible pipeline.

15. The manuscript presents different odds ratios for the primary association across the main text and sensitivity tables, and the distinction between models is not sufficiently clear. For example, the Results section reports the fully adjusted estimate for the primary outcome as OR = 0.84 (95% CI: 0.77–0.92) (lines 147–149), and Table 1 reports the same estimate for Model 3. However, Table 4 reports a different ‘Full sample’ estimate of OR = 0.82 (95% CI: 0.75–0.89) (Table 4, lines 699–704), with the note ‘Base model: age, sex, comorbidities, country’ (line 705). This differs from the sequential modelling strategy described in Methods (lines 110–116), where Models 1–3 are defined differently. Please clarify which covariates are included in each analysis, why the estimates differ, and which specification should be interpreted as the primary model.

This is fully resolved by the single-pipeline rebuild. There is now one clearly labelled primary model (confounder-only total association), one set of secondary (mediator-adjusted) models, and Table 4 uses the primary set. The note in each table specifies the exact covariates, and the previously divergent 0.82 vs 0.84 estimates no longer arise.
