# Supplementary material for: Informal care outside the household by older people and cardiovascular health of caregivers: A Cross-Sectional Analysis of the SHARE European study
Source: PLoS One. 2026 Oct 9;21(10):e0359074. doi: 10.1371/journal.pone.0359074 (PMC13652321; doi:10.1371/journal.pone.0359074)
Supplement: Supplementary file 3 [file pone.0359074.s005.pdf]

Response to Reviewers — PONE-D-26-04680R1 (Revised manuscript R2)

MS PONE-D-26-04680R1- Informal care outside the household by older people and cardiovascular health of caregivers: A Cross-Sectional Analysis of the SHARE European study

We thank the Academic Editor (Dr. Mickael Essouma) and both reviewers for their careful and constructive evaluation of our revised manuscript. The comments have further improved the clarity and accuracy of our work. Reviewer #2 recommends acceptance, and we have addressed every point raised by the Editor and by Reviewer #1 (Dr. Mahmoud A. Abdel-Fattah) in this second revision. Reviewer comments are shown in italics, followed by our response; line numbers refer to the revised manuscript with tracked changes.

Overarching note on this revision

In this revision we (i) relabelled the outcome consistently as "any self-reported cardiovascular disease" throughout, (ii) corrected the wording of the E-value conversion, (iii) renamed the exposure to "informal care outside the household" and revised the title, definition, abstract, keywords, and metadata accordingly, (iv) resolved the Figshare DOI and corrected the data-availability submission entry, (v) added a weighted sensitivity analysis, (vi) promoted the flow diagram to Figure 1, (vii) expanded the DAG with unmeasured confounders and inter-mediator arrows, (viii) revised the geographic effect-modification analysis to use a lower-dimensional, cluster-robust regional interaction, retaining the country-level analysis in S1 Table for transparency, and (ix) completed a final consistency check of all in-text estimates against the tables/figures. The substantive findings are unchanged: informal care remains associated with 15% lower odds of any self-reported cardiovascular disease (OR = 0.85, 95% CI: 0.78–0.93) in the confounder-adjusted model, which includes household wealth.

Response to the Academic Editor

Editor comment I — Inconsistent labelling of the "cardiovascular" endpoint

> *In lines 133 and 134, you mentioned that the primary outcome was ischemic heart disease, meanwhile below in the same paragraph, you clearly specify that the "cardiovascular" variable included not only ischemic heart disease, but also any other heart disease such as congestive heart failure, and you therefore labelled and interpreted the cardiovascular endpoint as IHD/heart problem. However, you did not consistently label the cardiovascular endpoint as IHD/heart problem throughout the manuscript… when going through lines 313 and 360, it seems that the "cardiovascular" endpoint did not just include IHD and any other*

heart disease, but also stroke (which is not a heart disease, but a [cerebro]vascular disease). To be fair, I therefore suggest labelling the "cardiovascular" endpoint as "any self-reported cardiovascular disease" throughout the manuscript, rather than "self-reported IHD" or "self-reported IHD/heart problem."

Response. We thank the Editor for this important point and agree fully. We have relabelled the endpoint as "any self-reported cardiovascular disease" throughout the manuscript (Abstract; Introduction; Materials and Methods, Outcomes section; Results, headings and body text; Discussion; Limitations; Tables 1–5 and their notes; and figure legends). The changes include the lines flagged by the Editor (Abstract; lines 134, 241, 243, 261, 282, 313, 327, 360, 412).

To remove the ambiguity the Editor identified, we now distinguish two endpoints explicitly in the Methods and Results:

1. **Primary outcome —** "self-reported cardiovascular disease (heart disease)." This corresponds to the single SHARE item *"Has a doctor ever told you that you had a heart attack, coronary thrombosis, coronary occlusion, or any other heart problem including congestive heart failure?"* We emphasise that this aggregates several cardiac conditions (including heart failure) and is not a clinically specific myocardial infarction endpoint; it also reflects lifetime rather than incident disease.
2. **Secondary outcome —** "any self-reported cardiovascular disease (including stroke)." This combined endpoint adds stroke to the primary outcome. We retain stroke as an exploratory outcome, given its greater susceptibility to reverse causality through disabling motor, cognitive, and communication sequelae.

The term **"IHD"** (and "MI") has been removed throughout except where we explain what was previously assumed and why the terminology was changed. The header of the Results section (lines 239–240) now reads "Association between informal care and self-reported cardiovascular disease."

Editor comment II — Odds-ratio-to-risk-ratio conversion for the E-value (lines 182–185)

You claimed "Because the outcome is common, we approximated the risk ratio from the odds ratio using the square-root transformation before computing the E-value, and we also report the value obtained by treating the odds ratio directly as a risk ratio." Wasn't it a mistake to

consider the odds ratio as an approximation of the risk ratio? Indeed, odds ratios are commonly considered approximations of risk ratios when the event is uncommon/rare, rather than when it is common.

Response. We thank the Editor and agree that our previous wording was unclear and could be misread. We have corrected the passage. The intended logic is the following: odds ratios approximate risk ratios only under the rare-outcome assumption; because our outcome was not rare (prevalence ≈ 12%), that assumption does not hold, and treating the odds ratio directly as a risk ratio would overstate the E-value. We therefore used the square-root transformation (RR ≈ √OR), the conversion recommended for common outcomes by VanderWeele (VanderWeele TJ, Ding P. *Sensitivity Analysis in Observational Research: Introducing the E-Value.* Ann Intern Med. 2017; see also the E-value guidance at https://www.evalue-calculator.com), to obtain an approximate risk ratio before computing the E-value. We also report the value obtained by treating the odds ratio directly as a risk ratio, but we now state explicitly that this latter value overstates robustness for a common outcome and is presented only for comparison.

The revised wording (Methods, Statistical analysis) reads:

Odds ratios approximate risk ratios only under the rare-outcome assumption. Because the outcome was not rare in our sample (prevalence ≈ 12%), that assumption does not hold; following VanderWeele and Ding (2017), we therefore used the square-root transformation (RR ≈ √OR) to obtain an approximate risk ratio before computing the E-value. We also report the E-value obtained by treating the odds ratio directly as a risk ratio, which overstates robustness for a common outcome and is presented only for comparison.

We have harmonised the corresponding Discussion paragraph (formerly lines 338–343) with this framing and confirmed that the previously requested deletion of the phrase *"comparable to many established lifestyle–disease associations"* has been carried through to the clean version.

Editor comment iii — Discretionary comments (exposure term, definition, and title)

The definition of "informal helping outside the household"... could fit with the definition of "informal (social) care," especially if the helpers were not paid to provide support, and if people who received support were identified as vulnerable individuals… I suggest adding

those details in the definition and eventually modifying the expression "informal helping outside the household" by "informal care outside the household" throughout the manuscript. Furthermore, it is unclear whether the cardiovascular health mentioned in the manuscript's title is about the caregiver or the care recipient. Altogether, I suggest modifying the current manuscript's title by a more precise one like: "Informal care outside the household by older people and cardiovascular health of caregivers: A Cross-Sectional Analysis of the SHARE European study."

Response. We thank the Editor and have adopted all three suggestions.

(a) **Exposure term.** We have replaced *"informal helping outside the household"* with "informal care outside the household" throughout the manuscript (title, short title, abstract, keywords, introduction, methods, results, discussion, table/figure legends) and in the submission metadata and cover letter.

(b) **Definition.** We have expanded the definition in both the Introduction and the Materials and Methods to align with the concept of informal (social) care, citing the source suggested by the Editor (PMC10391834):

In this study, "informal care outside the household" refers to the provision of practical, non-professional, and unpaid assistance (e.g., household tasks, transport, paperwork, repairs, or gardening) in the previous 12 months to relatives, friends, or neighbours who do not live in the respondent's household and who are frequently vulnerable individuals (e.g., with physical or mental disabilities, chronic illness, or frailty) needing extra help to carry out their daily activities. This is captured by the SHARE social-support module and is distinct from co-residential/within-household caregiving, which the literature links to different and often adverse health implications.

We add the following transparency note, because the SHARE item does not directly record whether helpers were paid or formally assess recipient vulnerability:

We note that the SHARE item does not directly record whether helpers were paid or formally assess recipient vulnerability; our exposure is therefore consistent with the construct of informal care outside the household as defined above, while recipient vulnerability and

unpaid status are inferred from the nature of the practical assistance and the non-co-residential relationship.

(c) **Title.** We have adopted the Editor's proposed title:

> **"Informal care outside the household by older people and cardiovascular health of caregivers: A Cross-Sectional Analysis of the SHARE European study."**

This title makes explicit that the cardiovascular health examined is that of the **caregiver**, not the care recipient, as the Editor requested. The title page, short title, abstract, author summary, keywords, cover letter, and submission-system metadata have all been updated accordingly.

Journal Requirements

1. **Recommended citations.** The Editor recommended evaluating the publication on informal care (PMC10391834). We reviewed it and judged it directly relevant to our exposure definition; we have therefore cited it in the Introduction and Materials and Methods (new reference [XX]) and in the reference list. No other reviewer-recommended citations were made in this round.

2. **Reference list —** completeness, correctness, and retracted articles. We have re-checked the full reference list for completeness and correct formatting. We confirm that none of the cited works has been retracted; to our knowledge no retraction notice applies to any of our references. Any change to the reference list (addition of reference [XX], PMC10391834) is reported here and reflected in the tracked-changes version.

Response to Reviewer #1 (Dr. Mahmoud A. Abdel-Fattah)

> *Reviewer #1: All comments have been addressed.*

We are grateful to Reviewer #1 for confirming that the previous round's comments were addressed and for the six additional refinements below.

Reviewer #1, Comment 1 — Data Availability Statement

The statement includes a placeholder [insert DOI once available / or available at: URL]. This should be resolved before final publication. The statement in the submission system, "Yes - all data are fully available without restriction," directly contradicts the detailed Data Availability Statement that follows, which clearly explains that the data are third-party SHARE data and cannot be redistributed by the authors.

Response. Addressed. (a) We have obtained and inserted the actual Figshare DOI for our complete, single-pipeline analysis code and codebook (data preparation → analysis → tables → figures), and the placeholder has been removed throughout. (b) We have corrected the submission-system entry to remove the contradiction. Because the SHARE data are owned by a third party and cannot be redistributed by the authors, we have not selected "Yes — all data are fully available without restriction." Instead, we have used the option for third-party / restricted-access data and explained that the SHARE data are freely available to all registered researchers for scientific purposes through the SHARE Research Data Centre (https://share-eric.eu/data/; SHARE Release 9.0.0), while our full analysis code and codebook are publicly deposited in Figshare with a citable DOI. The revised Data Availability Statement reads:

The data used in this study are owned by a third party (the Survey of Health, Ageing and Retirement in Europe, SHARE) and cannot be directly redistributed by the authors. The data are freely available to all registered researchers for scientific purposes through the SHARE Research Data Centre website (https://share-eric.eu/data/; SHARE Release 9.0.0). To ensure complete transparency and reproducibility, the full statistical and data-preparation code used for this study has been deposited in a public Figshare repository (DOI: 10.6084/m9.figshare.33122441).

Reviewer #1, Comment 2 — Survey weights

The authors state, "weighted estimates could be added if the editor prefers." While unweighted analysis is defensible given the within-population association aim, it would strengthen the manuscript to either conduct a sensitivity analysis using SHARE calibrated weights (if available), or more explicitly justify why unweighted analysis is appropriate.

Response. Addressed by adding the requested sensitivity analysis. We identified the SHARE Wave 9 calibrated cross-sectional individual weight (*cciw_w9*; calibrated to population totals by age, sex,

and region) and re-fitted the primary confounder-adjusted model — which includes household wealth — using these weights, with standard errors clustered at the country level. The weighted estimate (OR = 0.78; 95% CI: 0.63–0.96; E-value 1.53 using the square-root OR-to-RR conversion, and 1.90 treating the OR directly as an RR) remained in the same direction and statistically significant, and was if anything slightly more protective than the unweighted primary estimate. It is now reported in S1 Table. We retain the unweighted model as primary, because the research question concerns a within-population association rather than nationally representative prevalence, and we now state this rationale more explicitly in the Methods and Limitations, noting that the weighted sensitivity analysis confirms the robustness of inference.

Reviewer #1, Comment 3 — Promote the flow diagram

> *Since the flow diagram is essential for understanding the sample derivation, it should be promoted to a main figure.*

Response. Addressed. The participant flow diagram is now Figure 1, and all subsequent figures have been renumbered accordingly. All in-text references and the figure legends have been updated and cross-checked.

Reviewer #1, Comment 4 — DAG diagram

> *The paper's discussion acknowledges several plausible unmeasured confounders, yet these are not explicitly depicted in the DAG diagram. Adding them would enhance transparency. The DAG currently lists all mediators (BMI, smoking, etc.) as direct paths from the exposure to the outcome. However, in reality, these mediators influence each other (e.g., Depression → Physical Inactivity → BMI → Diabetes). Adding arrows between these mediators would make the causal structure more realistic.*

Response. Addressed. We have revised the DAG (now S2 Fig) in two ways:

(a) We added an explicit node for unmeasured confounders (baseline frailty/functional reserve, personality traits, cognitive status, healthcare utilisation, and broader socioeconomic position), with arrows into both the exposure and the outcome, reflecting the plausible residual confounding discussed in the Limitations.

(b) We added inter-mediator arrows to represent the causal dependencies among mediators, including Depression → Physical inactivity → BMI → Diabetes (and Hypertension downstream of BMI/physical inactivity), while preserving acyclicity and retaining the exposure→mediator→outcome paths. The figure legend now describes these additions and clarifies that the mediator-adjusted models estimate a controlled direct effect and may be affected by overadjustment bias.

Reviewer #1, Comment 5 — S1 Table

Please confirm S1 Table is included and complete.

Response. Confirmed. S1 Table is included and complete. It reports, for the primary model: McFadden's pseudo-R^2 (0.06), the c-statistic (0.68), the Hosmer–Lemeshow goodness-of-fit result; per-variable missingness (all < 1.3%; highest for household wealth, 1.2%; 269/63,150 = 0.43% excluded for missing data); the primary caring×region cluster-robust Wald test; the country-level interaction sensitivity analysis, with the rank-deficiency caveat for the cluster-robust covariance matrix; the pairs-cluster and wild-cluster bootstrap, population-averaged (GEE), and random-intercept estimates; and the newly added weighted sensitivity estimate.

Reviewer #1, Comment 6 — Final consistency check of figure/table references

The authors should conduct a final systematic check of all figure and table references against the final PDF or Word document to ensure consistency.

Response. Addressed. We performed a systematic pass against the final PDF and confirmed that (i) every estimate reported in the text matches the corresponding table/figure, because all numbers derive from the single reproducible pipeline; (ii) all "Figure ??" placeholders have been resolved and figure numbering updated after promoting the flow diagram to Figure 1; (iii) table notes specify the exact covariate set of each model; and (iv) the terminology change to "any self-reported cardiovascular disease" is applied consistently across text, tables, and legends. We provide both a tracked-changes version and a clean final version.

Additional internal corrections (reported for transparency)

During the final consistency check we also resolved three further items:

- **Author summary.** The *Author summary* heading was previously empty; PLOS ONE requires this section for research articles, and we have now completed it (plain-language summary of why and how the study was done, the main finding, and its interpretation as hypothesis-generating).
- **Financial disclosure.** We aligned the submission-system *Financial Disclosure* with the cover letter. The statement now reads: *"The authors received no specific funding for this work. EK is part-funded by the NIHR HealthTech Research Centre in Emergency and Acute Care (NIHR205301) and the Manchester British Heart Foundation Centre for Research Excellence (RE/24/130017). This work was also supported by a visiting research fellowship awarded by the University of Castilla-La Mancha."*
- **Geographic effect modification.** During final verification, we identified that the previously reported caring×country interaction statistic was not appropriate for the clustered inference used throughout the study. With 28 country clusters, a full country-level interaction requires 27 additional parameters and yields a rank-deficient cluster-robust covariance matrix. We therefore assessed effect modification using a five-region caring×region interaction with a cluster-robust Wald test. This interaction did not reach conventional statistical significance ($\chi^2(4) = 8.66$, $p = 0.070$). The country-level analysis is retained in S1 Table for transparency, together with the note that its cluster-robust covariance matrix is rank-deficient and therefore unsuitable for primary inference. Alternative regional classifications likewise did not yield statistically significant interactions.

Response to Reviewer #2

> *Reviewer #2: The authors have responded thoroughly to the review comments, and I recommend acceptance of the revised manuscript.*

We thank Reviewer #2 for the positive assessment and recommendation. No further points were raised; the present revision implements the Editor's and Reviewer #1's remaining requests while preserving the study's primary analytic framework and substantive conclusions.

We hope that these revisions satisfactorily address all remaining comments. We are grateful to the Editor and reviewers for their guidance, which has materially improved the manuscript, and we look forward to your decision.

Sincerely,

Raquel Cervigón & Evan Kontopantelis
