# Supplementary material for: Informal care outside the household by older people and cardiovascular health of caregivers: A Cross-Sectional Analysis of the SHARE European study
Source: PLoS One. 2026 Oct 9;21(10):e0359074. doi: 10.1371/journal.pone.0359074 (PMC13652321; doi:10.1371/journal.pone.0359074)
Supplement: Peer review history

# Peer review: Decision Letter 0

**DOI:** 10.1371/journal.pone.0359074.r001

**Authors:** Mickael Essouma

17 May 2026

Dear Dr. Cervigón,

Thank you for submitting your manuscript to PLOS ONE. After careful consideration, we feel that it has merit but does not fully meet PLOS ONE’s publication criteria as it currently stands. Therefore, we invite you to submit a revised version of the manuscript that addresses the points raised during the review process.

Please submit your revised manuscript by Jul 01 2026 11:59PM. If you will need more time than this to complete your revisions, please reply to this message or contact the journal office at plosone@plos.org. When you're ready to submit your revision, log on to https://www.editorialmanager.com/pone/ and select the 'Submissions Needing Revision' folder to locate your manuscript file.

- A letter that responds to each point raised by the academic editor and reviewer(s). You should upload this letter as a separate file labeled 'Response to Reviewers'.
- A marked-up copy of your manuscript that highlights changes made to the original version. You should upload this as a separate file labeled 'Revised Manuscript with Track Changes'.
- An unmarked version of your revised paper without tracked changes. You should upload this as a separate file labeled 'Manuscript'.

If you would like to make changes to your financial disclosure, please include your updated statement in your cover letter. Guidelines for resubmitting your figure files are available below the reviewer comments at the end of this letter.

If applicable, we recommend that you deposit your laboratory protocols in protocols.io to enhance the reproducibility of your results. Protocols.io assigns your protocol its own identifier (DOI) so that it can be cited independently in the future. For instructions see: https://journals.plos.org/plosone/s/submission-guidelines#loc-laboratory-protocols. Additionally, PLOS ONE offers an option for publishing peer-reviewed Lab Protocol articles, which describe protocols hosted on protocols.io. Read more information on sharing protocols at https://plos.org/protocols?utm_medium=editorial-email&utm_source=authorletters&utm_campaign=protocols.

As the corresponding author, your ORCID iD is verified in the submission system and will appear in the published article. PLOS supports the use of ORCID, and we encourage all coauthors to register for an ORCID iD and use it as well. Please encourage your coauthors to verify their ORCID iD within the submission system before final acceptance, as unverified ORCID iDs will not appear in the published article. *Only*  the individual author can complete the verification step; PLOS staff *cannot*  verify ORCID iDs on behalf of authors.

We look forward to receiving your revised manuscript.

Kind regards,

Mickael Essouma, M. D.

Academic Editor

PLOS One

Journal Requirements:

When submitting your revision, we need you to address these additional requirements.

1.Please ensure that your manuscript meets PLOS ONE's style requirements, including those for file naming. The PLOS ONE style templates can be found at

https://journals.plos.org/plosone/s/file?id=wjVg/PLOSOne_formatting_sample_main_body.pdf and

https://journals.plos.org/plosone/s/file?id=ba62/PLOSOne_formatting_sample_title_authors_affiliations.pdf

2. Thank you for uploading your study's underlying data set. Unfortunately, the repository you have noted in your Data Availability statement does not qualify as an acceptable data repository according to PLOS's standards.

At this time, please upload the minimal data set necessary to replicate your study's findings to a stable, public repository (such as figshare or Dryad) and provide us with the relevant URLs, DOIs, or accession numbers that may be used to access these data. For a list of recommended repositories and additional information on PLOS standards for data deposition, please see https://journals.plos.org/plosone/s/recommended-repositories.

3. When completing the data availability statement of the submission form, you indicated that you will make your data available on acceptance. We strongly recommend all authors decide on a data sharing plan before acceptance, as the process can be lengthy and hold up publication timelines. Please note that, though access restrictions are acceptable now, your entire data will need to be made freely accessible if your manuscript is accepted for publication. This policy applies to all data except where public deposition would breach compliance with the protocol approved by your research ethics board. If you are unable to adhere to our open data policy, please kindly revise your statement to explain your reasoning and we will seek the editor's input on an exemption. Please be assured that, once you have provided your new statement, the assessment of your exemption will not hold up the peer review process.

4. Please provide a complete Data Availability Statement in the submission form, ensuring you include all necessary access information or a reason for why you are unable to make your data freely accessible. If your research concerns only data provided within your submission, please write "All data are in the manuscript and/or supporting information files" as your Data Availability Statement.

5. If the reviewer comments include a recommendation to cite specific previously published works, please review and evaluate these publications to determine whether they are relevant and should be cited. There is no requirement to cite these works unless the editor has indicated otherwise.

Additional Editor Comments:

I think the authors should define the expression "Informal helping outside the household" in the introduction section and in the Materials and Methods sections.

Conform to PLOS ONE author guidelines when formatting the manuscript, eg when citing references in the text.

Consider revising the Materials and Methods section entirely conforming to STROBE guidelines (https://journals.plos.org/plosmedicine/article?id=10.1371/journal.pmed.0040297) and uploading a STROBE checklist to demonstrate compliance with STROBE guidelines. This entails subdividing the Materials and Methods section into many sub-sections such as Study design, setting and period, Study population/Data source (clear description of SHARE, inclusion and exclusion criteria, sampling method used, method of estimation of the sample size used), data collection (including who collected data, whether they were trained before collecting data, the data items that were collected, whether you did quality control of the data collected), exposure and outcome (with definition of the variable "Informal helping outside the household". As largely highlighted by Reviewer 2, there is an issue with the definition of the main outcome variable which is likely "all-cause CVD" rather than MI), statistical analysis (reporting following Reviewers' recommendations and SAMPL guidelines for statistical reporting in biomedical research https://onlinelibrary.wiley.com/doi/abs/10.1002/9781118715598.ch25), Ethical issues, and Patient and Public involvement.

Consider improving the readability of the results section by subdividing it into sub-sections as well. For example: Participant characteristics, Association between informal helping outside the household and all-cause CVD, Sensitivity analyses, Subgroup analyses. You would like to discuss the results in the Discussion section following the same plan.

Mickael Essouma, M.D.

[Note: HTML markup is below. Please do not edit.]

Reviewer's Responses to Questions

**Comments to the Author**

1. Is the manuscript technically sound, and do the data support the conclusions?

Reviewer #1: Partly

Reviewer #2: Partly

**********

2. Has the statistical analysis been performed appropriately and rigorously?

Reviewer #1: No

Reviewer #2: No

**********

3. Have the authors made all data underlying the findings in their manuscript fully available?

The PLOS Data policy

Reviewer #1: Yes

Reviewer #2: Yes

**********

4. Is the manuscript presented in an intelligible fashion and written in standard English?

Reviewer #1: Yes

Reviewer #2: Yes

**********

Reviewer #1: Journal: PLOS ONE

MS ID: PONE-D-26-04680

Title: Informal Helping Outside the Household and Cardiovascular Health in Older Europeans: A Cross-Sectional Analysis of SHARE

Version: 1 Date: 15 March 2026

Reviewer's report:

Comment 1—The manuscript provides a thorough description of the modelling strategy (multivariable logistic regression with country fixed effects and clustered standard errors) but fails to report any model diagnostics or fit statistics. This omission makes it impossible for the reader to assess the adequacy and stability of the statistical models upon which the primary conclusions rest. In any observational study, but particularly one with a large sample and multiple covariates, presenting only odds ratios and p-values is insufficient.

Comment 2—Some variables, like "depression" or "physical inactivity," could be mediators on the causal pathway between helping and cardiovascular health (i.e., helping improves mood and increases activity, which then reduces MI risk). Adjusting for mediators can bias the estimate of the total effect of helping, which is presumably what the authors are interested in. By including them in Models 2 and 3, the authors may be inadvertently "adjusting away" part of the very effect they are trying to measure.

Comment 3—The statement in the Results that helpers have lower odds of hypertension and diabetes, “which may partially explain the observed association with MI”, suggests they view these as mediators, yet they are treated as confounders in the main model. This is a conceptual inconsistency.

Comment 4—The authors must provide a clear rationale for their choice of covariates. The manuscript describes the covariates included in the models but provides no theoretical or empirical justification for why these specific variables were chosen and, more importantly, why others were not. If wealthier individuals are both healthier and more able to provide help (e.g., they have more resources, time, or social connections), then omitting wealth could confound the association.

Comment 5—By including country only as a fixed effect (to control for confounding), the authors are assuming the effect of helping is the same in every country. This is an assumption that should be tested.

Comment 6—The protective effect of helping might be stronger in countries with weaker formal social safety nets, where informal help is more critical, or weaker in countries with strong family cohesion norms. The authors should run a model with an interaction term between helping and country. A likelihood ratio test comparing models with and without the interaction term would formally assess if the effect varies across contexts.

Reviewer #2: This manuscript examines the association between informal helping outside the household and cardiovascular outcomes in older European adults using SHARE Wave 9 data. The topic is timely and potentially important, particularly given growing interest in social determinants of cardiovascular health and healthy ageing. The sample size is large and multinational, and the authors make a commendable effort to address reverse causality through several sensitivity analyses.

The manuscript is generally well written and clearly structured. However, there are important methodological and interpretative issues that substantially limit the strength of the conclusions. In its current form, I believe the manuscript requires substantial revision before it can be considered for publication. Primary concerns are outlined below, followed by additional specific comments.

Major comments

1. The principal limitation concerns the cross-sectional design and the interpretation of the findings. The exposure reflects helping behaviour during the previous 12 months, whereas the outcome is based on lifetime self-reported physician diagnosis of “heart attack, coronary thrombosis, coronary occlusion, or any other heart problem including congestive heart failure.” As a result, temporality is fundamentally unclear. Many cardiovascular events likely occurred years before the helping behaviour was measured. Although the authors acknowledge reverse causality and perform several exclusion analyses, these approaches cannot establish temporal ordering. The manuscript occasionally approaches causal interpretation despite repeated use of the word “association.” Statements suggesting that helping may “support cardiovascular prevention” or that encouraging helping engagement may confer cardiovascular benefits should therefore be softened substantially.

2. A major concern relates to outcome definition and validity. The manuscript repeatedly refers to “myocardial infarction” as the primary outcome, yet the SHARE item clearly includes a heterogeneous mixture of cardiovascular conditions, including congestive heart failure. This is not a clinically specific MI endpoint. The current terminology overstates the precision of the outcome measure and may mislead readers. The outcome should be relabelled throughout the manuscript using more accurate wording such as “self-reported ischemic heart disease/heart problems” or similar. This issue is especially important because the manuscript emphasises the clinical specificity of MI as a justification for the study design.

3. The covariate strategy requires more careful justification. Several variables included in the fully adjusted model may plausibly lie on the causal pathway between helping behaviour and cardiovascular health rather than functioning purely as confounders. For example, physical inactivity, depression, social network size, BMI, hypertension, and diabetes may partly mediate any relationship between social engagement and cardiovascular disease. Adjusting for potential mediators complicates interpretation of the fully adjusted odds ratios and may introduce overadjustment bias. The authors should explicitly discuss the assumed causal structure and clarify whether the intent is confounding control or estimation of a direct effect. A directed acyclic graph would strengthen the manuscript considerably.

4. The interpretation of the E-values is too strong. An E-value of 1.67 does not indicate robustness against unmeasured confounding to the degree implied in the Discussion. In observational social epidemiology, unmeasured factors such as baseline frailty, functional reserve, personality traits, cognitive status, healthcare utilisation, wealth, or broader socioeconomic position could plausibly have associations of this magnitude with both helping behaviour and cardiovascular disease. The E-value should therefore be interpreted more cautiously.

5. The reverse-causality analyses are interesting but should not be overstated. Excluding help recipients, participants with ADL limitations, and those with poor self-rated health does not eliminate the possibility that healthier individuals are more likely to help others. Moreover, such exclusions may introduce additional selection processes and complicate interpretation. The wording currently suggests that these analyses strongly support the robustness of the findings, whereas they more modestly indicate that the association persists in healthier subgroups.

6. The manuscript refers to a “dose-response relationship,” but the presented results do not convincingly demonstrate such a pattern. The odds ratio for helping one person is slightly stronger than for helping two or more people, and the confidence intervals overlap substantially. The data are more consistent with a threshold or participation effect than with a monotonic dose-response gradient. The text should be revised accordingly.

7. The statistical inference strategy warrants further discussion. The models use cluster-robust standard errors with only 25 country clusters. The authors appropriately note this limitation, but inference based on a relatively small number of clusters may still be unstable. Consideration of alternative approaches, such as wild-cluster bootstrap methods or multilevel modelling, would strengthen confidence in the results.

8. It is unclear whether survey weights were used. SHARE has a complex sampling structure, and failure to account for weights may affect representativeness and potentially bias estimates. The authors should explicitly state whether weights were applied and justify their choice.

9. The handling of missing data is insufficiently described. Although missingness is reportedly below 5%, the manuscript should provide the proportion missing for each variable and ideally compare characteristics of included versus excluded participants. Complete-case analysis may be reasonable, but more transparency is needed.

10. The subgroup analyses should be interpreted cautiously. The study may be underpowered to detect interaction effects despite the large overall sample, particularly because the observed associations are modest. The non-significant interaction tests do not necessarily imply absence of heterogeneity.

11. The manuscript would benefit from clearer separation between hypotheses and conclusions. At present, portions of the Discussion imply that helping behaviour itself may improve cardiovascular health. Given the limitations of the data, the findings are more appropriately interpreted as indicating that helping behaviour may be a marker of healthier ageing, better functional status, or broader social and behavioural advantages.

Other specific comments

1. Lines 58–68: the manuscript states multiple times (including in the Abstract), almost verbatim, that this is “the largest multinational study to date examining informal helping outside the household in relation to a clinically specific cardiovascular endpoint.” In addition to the unnecessary repetition, the description of the outcome as a “clinically specific cardiovascular endpoint” is difficult to justify given the outcome definition presented later in the Methods.

2. Lines 97–105: this is probably the most important methodological issue in the paper. The outcome question explicitly combines “heart attack,” “coronary thrombosis,” “coronary occlusion,” and “any other heart problem including congestive heart failure.” Therefore, the variable cannot reasonably be interpreted as a clinically specific myocardial infarction endpoint. I strongly recommend relabelling the outcome throughout the manuscript and moderating claims about clinical specificity.

3. Lines 110–116: the covariate adjustment strategy requires further conceptual justification. Several variables added in Models 2 and 3 (e.g., depression, physical inactivity, BMI, social network size, hypertension, diabetes) may plausibly act as mediators rather than confounders. The manuscript would benefit from a clearer explanation of the intended causal estimand and possibly a DAG.

4. Lines 118–121: the description of the E-value is technically correct, but later interpretation in the Discussion overstates its implications. An E-value of 1.67 is not especially large in observational social epidemiology and does not strongly exclude residual confounding.

5. Lines 122–130: the reverse-causality sensitivity analyses are thoughtful, but the manuscript should

acknowledge more explicitly that excluding participants with ADL limitations, poor health, or help receipt does not resolve temporality and may itself induce selection or collider bias.

6. Lines 147–149: the manuscript reports that helping was associated with “16% lower odds of MI.” Given the ambiguity of the outcome definition and the cross-sectional design, I suggest using more cautious wording such as “lower odds of self-reported cardiovascular disease/heart disease.”

7. Lines 152–158: the manuscript describes a “dose-response relationship,” but the estimates for helping one person (OR 0.81) and helping ≥2 persons (OR 0.84) are very similar and not monotonic. The findings appear more consistent with a threshold effect or simple participation effect than a true dose-response gradient.

8. Lines 168–180: the Discussion of reverse-causality probes is stronger than warranted. The statement that the “remarkable stability” of the estimates suggests the association is not fully explained by reverse causality should be softened. These exclusions mainly demonstrate persistence of association in progressively healthier subgroups.

9. Lines 203–206: the sentence interpreting the E-value should be moderated. The claim that the required degree of confounding is “comparable to many established lifestyle-disease associations” is subjective and may overstate robustness.

10. Lines 211–223: the manuscript again interprets the results in a quasi-causal manner (“engagement per se,” “underlies the association,” “encouraging helping engagement”). Given the study design, the conclusions should remain framed as hypothesis-generating associations.

11. Lines 241–242: the authors appropriately note that inference with 25 country clusters is “at the lower bound for reliable inference.” I would encourage them to consider supplementary analyses using alternative methods such as wild-cluster bootstrap standard errors or multilevel modelling to assess robustness.

12. Lines 244–249: this limitation deserves more prominence. The prevalence of the primary outcome is high partly because it reflects lifetime self-reported disease and partly because the question includes heart failure and other heart conditions. This should be emphasised earlier in the manuscript, not only in the limitations section.

13. Table 4: it would be helpful to clarify exactly which covariates are included in the “base model,” since the table note (“age, sex, comorbidities, country”) differs from the sequential modelling strategy described earlier.

14. Throughout the manuscript: please correct the remaining “Figure ??” placeholders and ensure consistency between estimates reported in text and tables.

15. The manuscript presents different odds ratios for the primary association across the main text and sensitivity tables, and the distinction between models is not sufficiently clear. For example, the Results section reports the fully adjusted estimate for the primary outcome as OR = 0.84 (95% CI: 0.77–0.92) (lines 147–149), and Table 1 reports the same estimate for Model 3. However, Table 4 reports a different ‘Full sample’ estimate of OR = 0.82 (95% CI: 0.75–0.89) (Table 4, lines 699–704), with the note ‘Base model: age, sex, comorbidities, country’ (line 705). This differs from the sequential modelling strategy described in Methods (lines 110–116), where Models 1–3 are defined differently. Please clarify which covariates are included in each analysis, why the estimates differ, and which specification should be interpreted as the primary model.

**********

what does this mean?). If published, this will include your full peer review and any attached files.

If you choose “no”, your identity will remain anonymous but your review may still be made public.

**Do you want your identity to be public for this peer review?**  For information about this choice, including consent withdrawal, please see our Privacy Policy

Reviewer #1: **Yes:**  Mahmoud A. Abdel-Fattah

Reviewer #2: No

**********

[NOTE: If reviewer comments were submitted as an attachment file, they will be attached to this email and accessible via the submission site. Please log into your account, locate the manuscript record, and check for the action link "View Attachments". If this link does not appear, there are no attachment files.]

To ensure your figures meet our technical requirements, please review our figure guidelines: https://journals.plos.org/plosone/s/figures

You may also use PLOS’s free figure tool, NAAS, to help you prepare publication quality figures: https://journals.plos.org/plosone/s/figures#loc-tools-for-figure-preparation.

NAAS will assess whether your figures meet our technical requirements by comparing each figure against our figure specifications.

# Author comment: Author response to Decision Letter 1

**DOI:** 10.1371/journal.pone.0359074.r002

30 Jun 2026

MS PONE-D-26-04680 — Informal Helping Outside the Household and Cardiovascular Health in Older Europeans: A Cross-Sectional Analysis of SHARE

We thank the Academic Editor and both reviewers for their careful and constructive evaluation. The comments have substantially improved the manuscript. Below we respond to each point in turn; manuscript line numbers refer to the revised version with tracked changes. Reviewer comments are shown in italics, followed by our response.

Overarching note on data, sample, and reproducibility.

In preparing this revision we rebuilt the entire analysis from the raw SHARE Wave 9 release through a single, fully scripted pipeline (data preparation → analysis → tables → figures), so that every number in the text, tables, and figures derives from one clearly defined analytical sample. The analytical sample comprises 62,881 community-dwelling respondents aged 50–84 from 28 countries, defined by complete data on the exposure, outcomes, and the variables used in our models.

We also took this opportunity to correct the analytical sample. The original submission inadvertently used a derived dataset that had been restricted to respondents with complete data on quality-of-life (CASP-12), loneliness, and personality measures — variables required for other SHARE-based projects but NOT used in the present study. Conditioning the sample on variables that are not part of the analysis excluded participants without justification and reduced the sample to 39,632. Because the present analysis does not use those variables, we now derive the sample directly from the full Wave 9 release and apply complete-case restriction only to the variables actually used in our models. This is the methodologically appropriate approach and increases the analytical sample to 62,881 without introducing selection on study-irrelevant variables. Reassuringly, the principal estimates are essentially unchanged (e.g., the primary association OR = 0.84), confirming that the substantive findings are robust; the larger sample improves precision and representativeness. This change also reconciles the numerical inconsistencies noted by Reviewer 2 (comments 13 and 15).

As part of this rebuild we also (i) relabelled the outcome, (ii) separated confounders from potential mediators and now report a confounder-only (total-association) model as primary, (iii) recomputed E-values using the appropriate odds-ratio-to-risk-ratio approximation, and (iv) added the model diagnostics, cross-country interaction test, and alternative-inference analyses requested below.

Response to the Academic Editor

1.Please ensure that your manuscript meets PLOS ONE's style requirements, including those for file naming. The PLOS ONE style templates can be found at

https://journals.plos.org/plosone/s/file?id=wjVg/PLOSOne_formatting_sample_main_body.pdf [journals.plos.org] and

https://journals.plos.org/plosone/s/file?id=ba62/PLOSOne_formatting_sample_title_authors_affiliations.pdf [track.editorialmanager.com]

We sincerely appreciate the opportunity to submit a revised version of our manuscript. We have thoroughly revamped our statistical modeling strategy using a causal framework, corrected the outcome variable labeling, and significantly toned down our interpretations to meet all of the journal's publication criteria.

2. Thank you for uploading your study's underlying data set. Unfortunately, the repository you have noted in your Data Availability statement does not qualify as an acceptable data repository according to PLOS's standards.

At this time, please upload the minimal data set necessary to replicate your study's findings to a stable, public repository (such as figshare or Dryad) and provide us with the relevant URLs, DOIs, or accession numbers that may be used to access these data. For a list of recommended repositories and additional information on PLOS standards for data deposition, please see https://journals.plos.org/plosone/s/recommended-repositories [track.editorialmanager.com].

We have generated and uploaded both required files.

3. When completing the data availability statement of the submission form, you indicated that you will make your data available on acceptance. We strongly recommend all authors decide on a data sharing plan before acceptance, as the process can be lengthy and hold up publication timelines. Please note that, though access restrictions are acceptable now, your entire data will need to be made freely accessible if your manuscript is accepted for publication. This policy applies to all data except where public deposition would breach compliance with the protocol approved by your research ethics board. If you are unable to adhere to our open data policy, please kindly revise your statement to explain your reasoning and we will seek the editor's input on an exemption. Please be assured that, once you have provided your new statement, the assessment of your exemption will not hold up the peer review process.

Thank you for this clarification regarding the open data policy. As noted in our manuscript, the data used in this study belong to a third party (Survey of Health, Ageing and Retirement in Europe - SHARE). Due to data protection regulations and the legal framework of the SHARE consortium, we do not have the right or ownership to publicly deposit the raw data or redistribute it ourselves.

However, these data are already fully and freely accessible to any researcher for scientific purposes. To comply with your policy while respecting third-party restrictions, we have revised our Data Availability Statement to explain exactly how readers can obtain the data, and we have publicly shared our complete analysis code to ensure full reproducibility.

Our revised Data Availability Statement is as follows:

"The data used in this study are owned by a third party (the Survey of Health, Ageing and Retirement in Europe, SHARE) and cannot be directly redistributed by the authors. The data are freely available to all registered researchers for scientific purposes through the SHARE Research Data Centre website (https://share-eric.eu/). The specific dataset used in this analysis corresponds to SHARE Release 9.0.0. To ensure complete transparency and reproducibility, the full statistical and data-preparation code used for this study has been deposited in a public Figshare repository [insert DOI once available / or available at: URL]."

4. Please provide a complete Data Availability Statement in the submission form, ensuring you include all necessary access information or a reason for why you are unable to make your data freely accessible. If your research concerns only data provided within your submission, please write "All data are in the manuscript and/or supporting information files" as your Data Availability Statement.

We have updated our Data Availability statement. Because SHARE data are owned by a third party and cannot be directly redistributed by us, we clarify that they are freely accessible to registered researchers through the SHARE Research Data Centre (release 9.0.0). Furthermore, to guarantee absolute reproducibility, we have deposited our complete, single-pipeline analysis code (data-preparation, analysis, and figure scripts) in a public Figshare repository with a citable DOI.

5. If the reviewer comments include a recommendation to cite specific previously published works, please review and evaluate these publications to determine whether they are relevant and should be cited. There is no requirement to cite these works unless the editor has indicated otherwise.

Thank you.

Additional Editor Comments:

I think the authors should define the expression "Informal helping outside the household" in the introduction section and in the Materials and Methods sections.

Conform to PLOS ONE author guidelines when formatting the manuscript, eg when citing references in the text.

We now define the exposure explicitly in both sections. In SHARE, this refers to practical help (e.g., household tasks, transport, paperwork, repairs, gardening) given in the previous 12 months to relatives, friends, or neighbours who do NOT live in the respondent’s household, captured by the SHARE social-support module. We state that this is distinct from co-residential/within-household care, which the literature links to different (often adverse) health implications, and which we deliberately exclude from the exposure.

Review Comments to the Author

Please use the space provided to explain your answers to the questions above. You may also include additional comments for the author, including concerns about dual publication, research ethics, or publication ethics. (Please upload your review as an attachment if it exceeds 20,000 characters)

Reviewer #1: Journal: PLOS ONE

MS ID: PONE-D-26-04680

Title: Informal Helping Outside the Household and Cardiovascular Health in Older Europeans: A Cross-Sectional Analysis of SHARE

Version: 1 Date: 15 March 2026

Reviewer's report:

Comment 1—The manuscript provides a thorough description of the modelling strategy (multivariable logistic regression with country fixed effects and clustered standard errors) but fails to report any model diagnostics or fit statistics. This omission makes it impossible for the reader to assess the adequacy and stability of the statistical models upon which the primary conclusions rest. In any observational study, but particularly one with a large sample and multiple covariates, presenting only odds ratios and p-values is insufficient.

We completely agree that model diagnostics are essential. In this revised version, we have calculated and reported the following diagnostic statistics for our primary model: McFadden's pseudo-R^2 (0.05), the area under the ROC curve (c-statistic = 0.68), and the Hosmer-Lemeshow goodness-of-fit test. These indicators have been integrated into the Results section and are detailed in the new Supporting Table S1. As expected for models utilizing broad social predictors, the discrimination is modest, which is now explicitly discussed. We are also now consistently reporting 95% confidence intervals for all estimates and prioritise discussing association sizes over statistical significance.

Comment 2—Some variables, like "depression" or "physical inactivity," could be mediators on the causal pathway between helping and cardiovascular health (i.e., helping improves mood and increases activity, which then reduces MI risk). Adjusting for mediators can bias the estimate of the total effect of helping, which is presumably what the authors are interested in. By including them in Models 2 and 3, the authors may be inadvertently "adjusting away" part of the very effect they are trying to measure.

We thank the reviewer for this crucial methodological point. We agree that including downstream mediators in the primary model introduces overadjustment bias. To resolve this, we mapped out our assumed causal structure using a Directed Acyclic Graph (DAG, S2 Fig). Our primary model has been changed to a total-association model, adjusting exclusively for true baseline confounders (age, sex, education, marital status, household wealth, and country). Analyses incorporating hypertension, diabetes, depression, BMI, smoking, and physical inactivity have been shifted to secondary analyses to estimate the "controlled direct effect," with explicit warnings to the reader about overadjustment.

Comment 3—The statement in the Results that helpers have lower odds of hypertension and diabetes, “which may partially explain the observed association with MI”, suggests they view these as mediators, yet they are treated as confounders in the main model. This is a conceptual inconsistency.

We agree. We now make the causal structure explicit with a directed acyclic graph (S2 Fig) and distinguish two estimands. The PRIMARY model is now a total-association model adjusting for confounders only (age, sex, education, marital status, household wealth, country). Models adding hypertension, diabetes, depression, BMI, smoking, and physical inactivity are presented as secondary analyses estimating a controlled direct effect, with the explicit caveat of possible overadjustment bias. We removed the sentence implying that lower hypertension/diabetes among helpers “explains” the association, since that framing treated mediators as confounders.

Comment 4—The authors must provide a clear rationale for their choice of covariates. The manuscript describes the covariates included in the models but provides no theoretical or empirical justification for why these specific variables were chosen and, more importantly, why others were not. If wealthier individuals are both healthier and more able to provide help (e.g., they have more resources, time, or social connections), then omitting wealth could confound the association.

We now justify each covariate by its assumed role in the DAG, and we have ADDED household wealth (SHARE household net worth, signed-log transform) to the confounder set, as the reviewer rightly notes that wealth may influence both helping and cardiovascular health. Its inclusion did not materially change the estimate.

Comment 5—By including country only as a fixed effect (to control for confounding), the authors are assuming the effect of helping is the same in every country. This is an assumption that should be tested.

Comment 6—The protective effect of helping might be stronger in countries with weaker formal social safety nets, where informal help is more critical, or weaker in countries with strong family cohesion norms. The authors should run a model with an interaction term between helping and country. A likelihood ratio test comparing models with and without the interaction term would formally assess if the effect varies across contexts.

Thank you for your comments. We tested this directly. Adding a helping×country interaction and comparing models by likelihood-ratio test showed no evidence of cross-country heterogeneity (LR χ² = 33.1, df = 26, p = 0.16). This is now reported in the Results and S1 Table, and we note that we were not powered to characterise country-specific modifiers such as welfare-regime strength.

Reviewer #2: This manuscript examines the association between informal helping outside the household and cardiovascular outcomes in older European adults using SHARE Wave 9 data. The topic is timely and potentially important, particularly given growing interest in social determinants of cardiovascular health and healthy ageing. The sample size is large and multinational, and the authors make a commendable effort to address reverse causality through several sensitivity analyses.

The manuscript is generally well written and clearly structured. However, there are important methodological and interpretative issues that substantially limit the strength of the conclusions. In its current form, I believe the manuscript requires substantial revision before it can be considered for publication. Primary concerns are outlined below, followed by additional specific comments.

Major comments

1. The principal limitation concerns the cross-sectional design and the interpretation of the findings. The exposure reflects helping behaviour during the previous 12 months, whereas the outcome is based on lifetime self-reported physician diagnosis of “heart attack, coronary thrombosis, coronary occlusion, or any other heart problem including congestive heart failure.” As a result, temporality is fundamentally unclear. Many cardiovascular events likely occurred years before the helping behaviour was measured. Although the authors acknowledge reverse causality and perform several exclusion analyses, these approaches cannot establish temporal ordering. The manuscript occasionally approaches causal interpretation despite repeated use of the word “association.” Statements suggesting that helping may “support cardiovascular prevention” or that encouraging helping engagement may confer cardiovascular benefits should therefore be softened substantially.

We have systematically softened the language. The Abstract, Results, and Discussion now frame findings as cross-sectional associations that are hypothesis-generating. We removed statements implying that helping “supports cardiovascular prevention” or confers benefits, and we state plainly that temporality cannot be established because the exposure is 12-month behaviour while the outcome is a lifetime self-reported diagnosis.

2. A major concern relates to outcome definition and validity. The manuscript repeatedly refers to “myocardial infarction” as the primary outcome, yet the SHARE item clearly includes a heterogeneous mixture of cardiovascular conditions, including congestive heart failure. This is not a clinically specific MI endpoint. The current terminology overstates the precision of the outcome measure and may mislead readers. The outcome should be relabelled throughout the manuscript using more accurate wording such as “self-reported ischemic heart disease/heart problems” or similar. This issue is especially important because the manuscript emphasises the clinical specificity of MI as a justification for the study design.

Addressed as above. The outcome is relabelled throughout as self-reported ischemic heart disease / heart problems, the questionnaire wording is quoted verbatim, and we explicitly acknowledge that it aggregates heart attack with other heart conditions including heart failure and is not a clinically specific MI endpoint. The “largest study of a clinically specific endpoint” claim has been removed.

3. The covariate strategy requires more careful justification. Several variables included in the fully adjusted model may plausibly lie on the causal pathway between helping behaviour and cardiovascular health rather than functioning purely as confounders. For example, physical inactivity, depression, social network size, BMI, hypertension, and diabetes may partly mediate any relationship between social engagement and cardiovascular disease. Adjusting for potential mediators complicates interpretation of the fully adjusted odds ratios and may introduce overadjustment bias. The authors should explicitly discuss the assumed causal structure and clarify whether the intent is confounding control or estimation of a direct effect. A directed acyclic graph would strengthen the manuscript considerably.

Addressed via the DAG (S2 Fig) and the confounder-only primary model described under Reviewer 1, comments 2–3. The intended estimand (total association) is now stated explicitly, with the mediator-adjusted models clearly labelled as secondary.

4. The interpretation of the E-values is too strong. An E-value of 1.67 does not indicate robustness against unmeasured confounding to the degree implied in the Discussion. In observational social epidemiology, unmeasured factors such as baseline frailty, functional reserve, personality traits, cognitive status, healthcare utilisation, wealth, or broader socioeconomic position could plausibly have associations of this magnitude with both helping behaviour and cardiovascular disease. The E-value should therefore be interpreted more cautiously.

We recomputed the E-value using the recommended odds-ratio-to-risk-ratio (square-root) approximation for a common outcome. For the primary estimate this yields E ≈ 1.4 (vs 1.67 when the OR is treated directly as a risk ratio); we now report both and state that confounding of this magnitude—from frailty, functional reserve, personality, cognition, healthcare use, or socioeconomic position—is entirely plausible, so the E-value does NOT provide strong reassurance. The “comparable to established lifestyle-disease associations” claim was deleted.

5. The reverse-causality analyses are interesting but should not be overstated. Excluding help recipients, participants with ADL limitations, and those with poor self-rated health does not eliminate the possibility that healthier individuals are more likely to help others. Moreover, such exclusions may introduce additional selection processes and complicate interpretation. The wording currently suggests that these analyses strongly support the robustness of the findings, whereas they more modestly indicate that the association persists in healthier subgroups.

We softened this throughout and now interpret the exclusions more carefully. The association is robust to excluding objective markers of poor health (help recipients, ADL limitations, depression), individually or combined. It attenuates only when excluding fair/poor self-rated health, but that measure is a broad construct reflecting mood and social participation as well as physical disease and removes a large, disease-enriched fraction of the sample, so conditioning on it may induce selection or collider bias. We state explicitly that none of these cross-sectional exclusions can establish temporality.

6. The manuscript refers to a “dose-response relationship,” but the presented results do not convincingly demonstrate such a pattern. The odds ratio for helping one person is slightly stronger than for helping two or more people, and the confidence intervals overlap substantially. The data are more consistent with a threshold or participation effect than with a monotonic dose-response gradient. The text should be revised accordingly.

We agree. With overlapping confidence intervals and a non-monotonic pattern (1 person vs ≥2 persons), we now describe the finding as a threshold/participation effect rather than a dose-response gradient.

7. The statistical inference strategy warrants further discussion. The models use cluster-robust standard errors with only 25 country clusters. The authors appropriately note this limitation, but inference based on a relatively small number of clusters may still be unstable. Consideration of alternative approaches, such as wild-cluster bootstrap methods or multilevel modelling, would strengthen confidence in the results.

We added robustness analyses: a pairs-cluster bootstrap and a wild-cluster bootstrap (boottest), and complementary population-averaged (GEE) and random-intercept multilevel models. All yielded estimates consistent with the primary model (OR ~0.84; S1 Table). These commands are included in the deposited analysis code.

8. It is unclear whether survey weights were used. SHARE has a complex sampling structure, and failure to account for weights may affect representativeness and potentially bias estimates. The authors should explicitly state whether weights were applied and justify their choice.

The analytical extract does not carry SHARE calibrated weights; analyses are therefore unweighted, and we account for the cross-national structure through country fixed effects and clustering. Because the aim is within-population association rather than nationally representative prevalence, an unweighted model is appropriate, and the GEE/multilevel robustness checks were consistent. This is now stated explicitly, and we note weighted estimates could be added if the editor prefers.

9. The handling of missing data is insufficiently described. Although missingness is reportedly below 5%, the manuscript should provide the proportion missing for each variable and ideally compare characteristics of included versus excluded participants. Complete-case analysis may be reasonable, but more transparency is needed.

We now report per-variable missingness and describe the complete-case construction (S1 Table). The analytical sample is defined by complete data on the exposure, outcomes, and model covariates only; as explained in the overarching note, we no longer condition on study-irrelevant variables (CASP-12, loneliness, personality) that had previously reduced the sample. Missingness was below 1.3 percent for every model variable (highest for household wealth at 1.2 percent), and only 269 of 63,150 eligible respondents (0.43 percent) were excluded for missing data; the participant flow diagram (S1 Fig) shows the numbers excluded at each step.

10. The subgroup analyses should be interpreted cautiously. The study may be underpowered to detect interaction effects despite the large overall sample, particularly because the observed associations are modest. The non-significant interaction tests do not necessarily imply absence of heterogeneity.

We now caution that non-significant interaction tests do not establish the absence of effect modification and may reflect limited power, and we present subgroup results as exploratory.

11. The manuscript would benefit from clearer separation between hypotheses and conclusions. At present, portions of the Discussion imply that helping behaviour itself may improve cardiovascular health. Given the limitations of the data, the findings are more appropriately interpreted as indicating that helping behaviour may be a marker of healthier ageing, better functional status, or broader social and behavioural advantages.

The Discussion now frames the central finding as helping being a possible marker of healthier ageing / better functional status, rather than a cause of better cardiovascular health, and the Conclusions are explicitly hypothesis-generating.

Other specific comments

1. Lines 58–68: the manuscript states multiple times (including in the Abstract), almost verbatim, that this is “the largest multinational study to date examining informal helping outside the household in relation to a clinically specific cardiovascular endpoint.” In addition to the unnecessary repetition, the description of the outcome as a “clinically specific cardiovascular endpoint” is difficult to justify given the outcome definition presented later in the Methods.

The duplicated sentence has been removed, and the “clinically specific endpoint” characterisation has been deleted given the outcome definition.

2. Lines 97–105: this is probably the most important methodological issue in the paper. The outcome question explicitly combines “heart attack,” “coronary thrombosis,” “coronary occlusion,” and “any other heart problem including congestive heart failure.” Therefore, the variable cannot reasonably be interpreted as a clinically specific myocardial infarction endpoint. I strongly recommend relabelling the outcome throughout the manuscript and moderating claims about clinical specificity.

Agreed; this is the key change. The outcome is relabelled throughout and claims of clinical specificity are moderated, as detailed in major comment 2.

3. Lines 110–116: the covariate adjustment strategy requires further conceptual justification. Several variables added in Models 2 and 3 (e.g., depression, physical inactivity, BMI, social network size, hypertension, diabetes) may plausibly act as mediators rather than confounders. The manuscript would benefit from a clearer explanation of the intended causal estimand and possibly a DAG.

Addressed via the DAG and the confounder-only primary model (Reviewer 1, comments 2–3).

4. Lines 118–121: the description of the E-value is technically correct, but later interpretation in the Discussion overstates its implications. An E-value of 1.67 is not especially large in observational social epidemiology and does not strongly exclude residual confounding.

Recomputed and re-interpreted cautiously (major comment 4).

5. Lines 122–130: the reverse-causality sensitivity analyses are thoughtful, but the manuscript should

acknowledge more explicitly that excluding participants with ADL limitations, poor health, or help receipt does not resolve temporality and may itself induce selection or collider bias.

We now state this explicitly in the Methods and Discussion.

6. Lines 147–149: the manuscript reports that helping was associated with “16% lower odds of MI.” Given the ambiguity of the outcome definition and the cross-sectional design, I suggest using more cautious wording such as “lower odds of self-reported cardiovascular disease/heart disease.”

Reworded to “lower odds of self-reported ischemic heart disease.”

7. Lines 152–158: the manuscript describes a “dose-response relationship,” but the estimates for helping one person (OR 0.81) and helping ≥2 persons (OR 0.84) are very similar and not monotonic. The findings appear more consistent with a threshold effect or simple participation effect than a true dose-response gradient.

Reframed as a threshold/participation effect (major comment 6).

8. Lines 168–180: the Discussion of reverse-causality probes is stronger than warranted. The statement that the “remarkable stability” of the estimates suggests the association is not fully explained by reverse causality should be softened. These exclusions mainly demonstrate persistence of association in progressively healthier subgroups.

Softened to “the association persists in progressively healthier subgroups.”

9. Lines 203–206: the sentence interpreting the E-value should be moderated. The claim that the required degree of confounding is “comparable to many established lifestyle-disease associations” is subjective and may overstate robustness.

The “comparable to established lifestyle-disease associations” claim was removed.

10. Lines 211–223: the manuscript again interprets the results in a quasi-causal manner (“engagement per se,” “underlies the association,” “encouraging helping engagement”). Given the study design, the conclusions should remain framed as hypothesis-generating associations.

These phrasings were removed or reframed as associations.

11. Lines 241–242: the authors appropriately note that inference with 25 country clusters is “at the lower bound for reliable inference.” I would encourage them to consider supplementary analyses using alternative methods such as wild-cluster bootstrap standard errors or multilevel modelling to assess robustness.

Added bootstrap, GEE, and multilevel analyses (major comment 7).

12. Lines 244–249: this limitation deserves more prominence. The prevalence of the primary outcome is high partly because it reflects lifetime self-reported disease and partly because the question includes heart failure and other heart conditions. This should be emphasised earlier in the manuscript, not only in the limitations section.

We now flag the lifetime/heterogeneous nature of the outcome in the Methods (outcome definition) as well as in the Limitations.

13. Table 4: it would be helpful to clarify exactly which covariates are included in the “base model,” since the table note (“age, sex, comorbidities, country”) differs from the sequential modelling strategy described earlier.

Table 4 now uses, and its note specifies, the same confounder set as the primary total-association model, so the full-sample estimate matches the primary estimate.

14. Throughout the manuscript: please correct the remaining “Figure ??” placeholders and ensure consistency between estimates reported in text and tables.

All cross-references are fixed, and every estimate in the text now matches the corresponding table, because all numbers derive from the single reproducible pipeline.

15. The manuscript presents different odds ratios for the primary association across the main text and sensitivity tables, and the distinction between models is not sufficiently clear. For example, the Results section reports the fully adjusted estimate for the primary outcome as OR = 0.84 (95% CI: 0.77–0.92) (lines 147–149), and Table 1 reports the same estimate for Model 3. However, Table 4 reports a different ‘Full sample’ estimate of OR = 0.82 (95% CI: 0.75–0.89) (Table 4, lines 699–704), with the note ‘Base model: age, sex, comorbidities, country’ (line 705). This differs from the sequential modelling strategy described in Methods (lines 110–116), where Models 1–3 are defined differently. Please clarify which covariates are included in each analysis, why the estimates differ, and which specification should be interpreted as the primary model.

This is fully resolved by the single-pipeline rebuild. There is now one clearly labelled primary model (confounder-only total association), one set of secondary (mediator-adjusted) models, and Table 4 uses the primary set. The note in each table specifies the exact covariates, and the previously divergent 0.82 vs 0.84 estimates no longer arise.

Attachment Submitted filename: Response to Reviewers.pdf

# Peer review: Decision Letter 1

**DOI:** 10.1371/journal.pone.0359074.r003

**Authors:** Mickael Essouma

12 Jul 2026

Dear Dr. Cervigón,

Thank you for submitting your manuscript to PLOS ONE. After careful consideration, we feel that it has merit but does not fully meet PLOS ONE’s publication criteria as it currently stands. Therefore, we invite you to submit a revised version of the manuscript that addresses the points raised during the review process.

Please submit your revised manuscript by Aug 26 2026 11:59PM. If you will need more time than this to complete your revisions, please reply to this message or contact the journal office at plosone@plos.org. When you're ready to submit your revision, log on to https://www.editorialmanager.com/pone/ and select the 'Submissions Needing Revision' folder to locate your manuscript file.

- A letter that responds to each point raised by the academic editor and reviewer(s). You should upload this letter as a separate file labeled 'Response to Reviewers'.
- A marked-up copy of your manuscript that highlights changes made to the original version. You should upload this as a separate file labeled 'Revised Manuscript with Track Changes'.
- An unmarked version of your revised paper without tracked changes. You should upload this as a separate file labeled 'Manuscript'.

If you would like to make changes to your financial disclosure, please include your updated statement in your cover letter. Guidelines for resubmitting your figure files are available below the reviewer comments at the end of this letter.

If applicable, we recommend that you deposit your laboratory protocols in protocols.io to enhance the reproducibility of your results. Protocols.io assigns your protocol its own identifier (DOI) so that it can be cited independently in the future. For instructions see: https://journals.plos.org/plosone/s/submission-guidelines#loc-laboratory-protocols. Additionally, PLOS ONE offers an option for publishing peer-reviewed Lab Protocol articles, which describe protocols hosted on protocols.io. Read more information on sharing protocols at https://plos.org/protocols?utm_medium=editorial-email&utm_source=authorletters&utm_campaign=protocols.

As the corresponding author, your ORCID iD is verified in the submission system and will appear in the published article. PLOS supports the use of ORCID, and we encourage all coauthors to register for an ORCID iD and use it as well. Please encourage your coauthors to verify their ORCID iD within the submission system before final acceptance, as unverified ORCID iDs will not appear in the published article. *Only*  the individual author can complete the verification step; PLOS staff *cannot*  verify ORCID iDs on behalf of authors.

We look forward to receiving your revised manuscript.

Kind regards,

Mickael Essouma, M. D.

Academic Editor

PLOS One

**Journal Requirements:**

1. If the reviewer comments include a recommendation to cite specific previously published works, please review and evaluate these publications to determine whether they are relevant and should be cited. There is no requirement to cite these works unless the editor has indicated otherwise.

2. Please review your reference list to ensure that it is complete and correct. If you have cited papers that have been retracted, please include the rationale for doing so in the manuscript text, or remove these references and replace them with relevant current references. Any changes to the reference list should be mentioned in the rebuttal letter that accompanies your revised manuscript. If you need to cite a retracted article, indicate the article’s retracted status in the References list and also include a citation and full reference for the retraction notice.

**Additional Editor Comments:**

Editor's comments alongside reviewers' comments

I have the following remaining comments:

i) In lines 133 and 134, you mentioned that the primary outcome was ischemic heart disease, meanwhile below in the same paragraph, you clearly specify that the "cardiovascular" variable included not only ischemic heart disease, but also any other heart disease such as congestive heart failure, and you therefore labelled and interpreted the cardiovascular endpoint as IHD/heart problem. However, you did not consistently label the cardiovascular endpoint as IHD/heart problem throughout the manuscript. In the abstract, in lines 134, 241, 243, 261, 282, 313, 327, 360, and 412, the "cardiovascular" endpoint was labelled as "IHD". Furthermore, when going through lines 313 and 360, it seems that the "cardiovascular" endpoint did not just include IHD and any other heart disease, but also stroke (which is not a heart disease, but a [cerebro]vascular disease). To be fair, I therefore suggest labelling the "cardiovascular" endpoint as "any self-reported cardiovascular disease" throughout the manuscript, rather than "self-reported IHD" or "self-reported IHD/heart problem".

ii) In lines 182 to 185, you claimed "Because the outcome is common, we approximated the risk ratio from the odds ratio using the square-root transformation before computing the E-value, and we also report the value obtained by treating the odds ratio directly as a risk ratio.". Wasn't it a mistake to consider the odds ratio as an approximation of the risk ratio? Indeed, odds ratios are commonly considered approximations of risk ratios when the event is uncommon/rare, rather than when it is common: see https://ourworldindata.org/risk-ratios-odds-ratios-risk-differences-how-do-researchers-calculate-the-risk-from-a-risk-factor. Consider addressing this issue.

iii) Discretionary comments. The definition of “informal helping outside the household" provided in the introduction and Materials and Methods section of the revised manuscript could fit with the definition of "informal (social) care", especially if the helpers were not paid to provide support, and if people who received support were identified as vulnerable individuals (with physical/mental disabilities, illnesses...) needing extra-help to carry out their daily activities (https://pmc.ncbi.nlm.nih.gov/articles/PMC10391834/). I suggest adding those details in the definition and eventually modifying the expression “informal helping outside the household" by "informal care outside the household" throughout the manuscript. Furthermore, it is unclear whether the cardiovascular health mentioned in the manuscript’s title is about the caregiver or the care recipient. Altogether, I suggest modifying the current manuscript’s title by a more precise one like: "Informal care outside the household by older people and cardiovascular health of caregivers: A Cross-Sectional Analysis of the SHARE European study".

Mickael Essouma, M.D.

[Note: HTML markup is below. Please do not edit.]

Reviewers' comments:

Reviewer's Responses to Questions

**Comments to the Author**

Reviewer #1: All comments have been addressed

Reviewer #2: All comments have been addressed

**********

2. Is the manuscript technically sound, and do the data support the conclusions?

Reviewer #1: Yes

Reviewer #2: Yes

**********

3. Has the statistical analysis been performed appropriately and rigorously?

Reviewer #1: Yes

Reviewer #2: Yes

**********

4. Have the authors made all data underlying the findings in their manuscript fully available?

The PLOS Data policy

Reviewer #1: No

Reviewer #2: Yes

**********

5. Is the manuscript presented in an intelligible fashion and written in standard English?

Reviewer #1: Yes

Reviewer #2: Yes

**********

**Reviewer #1:**  Journal: PLOS ONE

MS ID: PONE-D-26-04680R1

Title: Informal Helping Outside the Household and Cardiovascular Health in Older Europeans: A Cross-Sectional Analysis of SHARE

Version: R1 Date: 11 July 2026

Reviewer's report:

Comment 1—Data availability statement:

• The statement includes a placeholder [insert DOI once available / or available at: URL]. This should be resolved before final publication.

• The statement in the submission system, “Yes - all data are fully available without restriction,” directly contradicts the detailed Data Availability Statement that follows, which clearly explains that the data are third-party SHARE data and cannot be redistributed by the authors.

Comment 2—The authors state, “weighted estimates could be added if the editor prefers.” While unweighted analysis is defensible given the within-population association aim, it would strengthen the manuscript to either:

• Conduct a sensitivity analysis using SHARE calibrated weights (if available), or

• More explicitly justify why unweighted analysis is appropriate for the research question (currently stated but brief).

Comment 3—Since the flow diagram is essential for understanding the sample derivation, it should be promoted to a main figure.

Comment 4—DAG diagram:

• The paper's discussion acknowledges several plausible unmeasured confounders, yet these are not explicitly depicted in the DAG diagram. Adding them would enhance transparency.

• The DAG currently lists all mediators (BMI, smoking, etc.) as direct paths from the exposure to the outcome. However, in reality, these mediators influence each other (e.g., Depression → Physical Inactivity → BMI → Diabetes). Adding arrows between these mediators would make the causal structure more realistic.

Comment 5—Please confirm S1 Table is included and complete.

Comment 6—The authors should conduct a final systematic check of all figure and table references against the final PDF or Word document to ensure consistency.

**Reviewer #2:**  The authors have responded thoroughly to the review comments, and I recommend acceptance of the revised manuscript.

**********

what does this mean?). If published, this will include your full peer review and any attached files.

If you choose “no”, your identity will remain anonymous but your review may still be made public.

**Do you want your identity to be public for this peer review?**  For information about this choice, including consent withdrawal, please see our Privacy Policy

Reviewer #1: **Yes:**  Mahmoud A. Abdel-Fattah

Reviewer #2: No

**********

[NOTE: If reviewer comments were submitted as an attachment file, they will be attached to this email and accessible via the submission site. Please log into your account, locate the manuscript record, and check for the action link "View Attachments". If this link does not appear, there are no attachment files.]

To ensure your figures meet our technical requirements, please review our figure guidelines: https://journals.plos.org/plosone/s/figures

You may also use PLOS’s free figure tool, NAAS, to help you prepare publication quality figures: https://journals.plos.org/plosone/s/figures#loc-tools-for-figure-preparation.

NAAS will assess whether your figures meet our technical requirements by comparing each figure against our figure specifications.

# Author comment: Author response to Decision Letter 2

**DOI:** 10.1371/journal.pone.0359074.r004

6 Sep 2026

Response to Reviewers — PONE-D-26-04680R1 (Revised manuscript R2)

MS PONE-D-26-04680R1- Informal care outside the household by older people and cardiovascular health of caregivers: A Cross-Sectional Analysis of the SHARE European study

We thank the Academic Editor (Dr. Mickael Essouma) and both reviewers for their careful and constructive evaluation of our revised manuscript. The comments have further improved the clarity and accuracy of our work. Reviewer #2 recommends acceptance, and we have addressed every point raised by the Editor and by Reviewer #1 (Dr. Mahmoud A. Abdel-Fattah) in this second revision. Reviewer comments are shown in italics, followed by our response; line numbers refer to the revised manuscript with tracked changes.

Overarching note on this revision

In this revision we (i) relabelled the outcome consistently as "any self-reported cardiovascular disease" throughout, (ii) corrected the wording of the E-value conversion, (iii) renamed the exposure to "informal care outside the household" and revised the title, definition, abstract, keywords, and metadata accordingly, (iv) resolved the Figshare DOI and corrected the data-availability submission entry, (v) added a weighted sensitivity analysis, (vi) promoted the flow diagram to Figure 1, (vii) expanded the DAG with unmeasured confounders and inter-mediator arrows, (viii) revised the geographic effect-modification analysis to use a lower-dimensional, cluster-robust regional interaction, retaining the country-level analysis in S1 Table for transparency, and (ix) completed a final consistency check of all in-text estimates against the tables/figures. The substantive findings are unchanged: informal care remains associated with 15% lower odds of any self-reported cardiovascular disease (OR = 0.85, 95% CI: 0.78–0.93) in the confounder-adjusted model, which includes household wealth.

Response to the Academic Editor

Editor comment I — Inconsistent labelling of the "cardiovascular" endpoint

In lines 133 and 134, you mentioned that the primary outcome was ischemic heart disease, meanwhile below in the same paragraph, you clearly specify that the "cardiovascular" variable included not only ischemic heart disease, but also any other heart disease such as congestive heart failure, and you therefore labelled and interpreted the cardiovascular endpoint as IHD/heart problem. However, you did not consistently label the cardiovascular endpoint as IHD/heart problem throughout the manuscript… when going through lines 313 and 360, it seems that the "cardiovascular" endpoint did not just include IHD and any other heart disease, but also stroke (which is not a heart disease, but a [cerebro]vascular disease). To be fair, I therefore suggest labelling the "cardiovascular" endpoint as "any self-reported cardiovascular disease" throughout the manuscript, rather than "self-reported IHD" or "self-reported IHD/heart problem."

Response. We thank the Editor for this important point and agree fully. We have relabelled the endpoint as "any self-reported cardiovascular disease" throughout the manuscript (Abstract; Introduction; Materials and Methods, Outcomes section; Results, headings and body text; Discussion; Limitations; Tables 1–5 and their notes; and figure legends). The changes include the lines flagged by the Editor (Abstract; lines 134, 241, 243, 261, 282, 313, 327, 360, 412).

To remove the ambiguity the Editor identified, we now distinguish two endpoints explicitly in the Methods and Results:

1. Primary outcome — "self-reported cardiovascular disease (heart disease)." This corresponds to the single SHARE item "Has a doctor ever told you that you had a heart attack, coronary thrombosis, coronary occlusion, or any other heart problem including congestive heart failure?" We emphasise that this aggregates several cardiac conditions (including heart failure) and is not a clinically specific myocardial infarction endpoint; it also reflects lifetime rather than incident disease.

2. Secondary outcome — "any self-reported cardiovascular disease (including stroke)." This combined endpoint adds stroke to the primary outcome. We retain stroke as an exploratory outcome, given its greater susceptibility to reverse causality through disabling motor, cognitive, and communication sequelae.

The term "IHD" (and "MI") has been removed throughout except where we explain what was previously assumed and why the terminology was changed. The header of the Results section (lines 239–240) now reads "Association between informal care and self-reported cardiovascular disease."

Editor comment II — Odds-ratio-to-risk-ratio conversion for the E-value (lines 182–185)

You claimed "Because the outcome is common, we approximated the risk ratio from the odds ratio using the square-root transformation before computing the E-value, and we also report the value obtained by treating the odds ratio directly as a risk ratio." Wasn't it a mistake to consider the odds ratio as an approximation of the risk ratio? Indeed, odds ratios are commonly considered approximations of risk ratios when the event is uncommon/rare, rather than when it is common.

Response. We thank the Editor and agree that our previous wording was unclear and could be misread. We have corrected the passage. The intended logic is the following: odds ratios approximate risk ratios only under the rare-outcome assumption; because our outcome was not rare (prevalence ≈ 12%), that assumption does not hold, and treating the odds ratio directly as a risk ratio would overstate the E-value. We therefore used the square-root transformation (RR ≈ √OR), the conversion recommended for common outcomes by VanderWeele (VanderWeele TJ, Ding P. Sensitivity Analysis in Observational Research: Introducing the E-Value. Ann Intern Med. 2017; see also the E-value guidance at https://www.evalue-calculator.com), to obtain an approximate risk ratio before computing the E-value. We also report the value obtained by treating the odds ratio directly as a risk ratio, but we now state explicitly that this latter value overstates robustness for a common outcome and is presented only for comparison.

The revised wording (Methods, Statistical analysis) reads:

Odds ratios approximate risk ratios only under the rare-outcome assumption. Because the outcome was not rare in our sample (prevalence ≈ 12%), that assumption does not hold; following VanderWeele and Ding (2017), we therefore used the square-root transformation (RR ≈ √OR) to obtain an approximate risk ratio before computing the E-value. We also report the E-value obtained by treating the odds ratio directly as a risk ratio, which overstates robustness for a common outcome and is presented only for comparison.

We have harmonised the corresponding Discussion paragraph (formerly lines 338–343) with this framing and confirmed that the previously requested deletion of the phrase "comparable to many established lifestyle–disease associations" has been carried through to the clean version.

Editor comment iii — Discretionary comments (exposure term, definition, and title)

The definition of "informal helping outside the household"… could fit with the definition of "informal (social) care," especially if the helpers were not paid to provide support, and if people who received support were identified as vulnerable individuals… I suggest adding those details in the definition and eventually modifying the expression "informal helping outside the household" by "informal care outside the household" throughout the manuscript. Furthermore, it is unclear whether the cardiovascular health mentioned in the manuscript's title is about the caregiver or the care recipient. Altogether, I suggest modifying the current manuscript's title by a more precise one like: "Informal care outside the household by older people and cardiovascular health of caregivers: A Cross-Sectional Analysis of the SHARE European study."

Response. We thank the Editor and have adopted all three suggestions.

(a) Exposure term. We have replaced "informal helping outside the household" with "informal care outside the household" throughout the manuscript (title, short title, abstract, keywords, introduction, methods, results, discussion, table/figure legends) and in the submission metadata and cover letter.

(b) Definition. We have expanded the definition in both the Introduction and the Materials and Methods to align with the concept of informal (social) care, citing the source suggested by the Editor (PMC10391834):

In this study, "informal care outside the household" refers to the provision of practical, non-professional, and unpaid assistance (e.g., household tasks, transport, paperwork, repairs, or gardening) in the previous 12 months to relatives, friends, or neighbours who do not live in the respondent's household and who are frequently vulnerable individuals (e.g., with physical or mental disabilities, chronic illness, or frailty) needing extra help to carry out their daily activities. This is captured by the SHARE social-support module and is distinct from co-residential/within-household caregiving, which the literature links to different and often adverse health implications.

We add the following transparency note, because the SHARE item does not directly record whether helpers were paid or formally assess recipient vulnerability:

We note that the SHARE item does not directly record whether helpers were paid or formally assess recipient vulnerability; our exposure is therefore consistent with the construct of informal care outside the household as defined above, while recipient vulnerability and unpaid status are inferred from the nature of the practical assistance and the non-co-residential relationship.

(c) Title. We have adopted the Editor's proposed title:

"Informal care outside the household by older people and cardiovascular health of caregivers: A Cross-Sectional Analysis of the SHARE European study."

This title makes explicit that the cardiovascular health examined is that of the caregiver, not the care recipient, as the Editor requested. The title page, short title, abstract, author summary, keywords, cover letter, and submission-system metadata have all been updated accordingly.

Journal Requirements

1. Recommended citations. The Editor recommended evaluating the publication on informal care (PMC10391834). We reviewed it and judged it directly relevant to our exposure definition; we have therefore cited it in the Introduction and Materials and Methods (new reference [XX]) and in the reference list. No other reviewer-recommended citations were made in this round.

2. Reference list — completeness, correctness, and retracted articles. We have re-checked the full reference list for completeness and correct formatting. We confirm that none of the cited works has been retracted; to our knowledge no retraction notice applies to any of our references. Any change to the reference list (addition of reference [XX], PMC10391834) is reported here and reflected in the tracked-changes version.

Response to Reviewer #1 (Dr. Mahmoud A. Abdel-Fattah)

Reviewer #1: All comments have been addressed.

We are grateful to Reviewer #1 for confirming that the previous round's comments were addressed and for the six additional refinements below.

Reviewer #1, Comment 1 — Data Availability Statement

The statement includes a placeholder [insert DOI once available / or available at: URL]. This should be resolved before final publication. The statement in the submission system, "Yes - all data are fully available without restriction," directly contradicts the detailed Data Availability Statement that follows, which clearly explains that the data are third-party SHARE data and cannot be redistributed by the authors.

Response. Addressed. (a) We have obtained and inserted the actual Figshare DOI for our complete, single-pipeline analysis code and codebook (data preparation → analysis → tables → figures), and the placeholder has been removed throughout. (b) We have corrected the submission-system entry to remove the contradiction. Because the SHARE data are owned by a third party and cannot be redistributed by the authors, we have not selected "Yes — all data are fully available without restriction." Instead, we have used the option for third-party / restricted-access data and explained that the SHARE data are freely available to all registered researchers for scientific purposes through the SHARE Research Data Centre (https://share-eric.eu/data/; SHARE Release 9.0.0), while our full analysis code and codebook are publicly deposited in Figshare with a citable DOI. The revised Data Availability Statement reads:

The data used in this study are owned by a third party (the Survey of Health, Ageing and Retirement in Europe, SHARE) and cannot be directly redistributed by the authors. The data are freely available to all registered researchers for scientific purposes through the SHARE Research Data Centre website (https://share-eric.eu/data/; SHARE Release 9.0.0). To ensure complete transparency and reproducibility, the full statistical and data-preparation code used for this study has been deposited in a public Figshare repository (DOI: 10.6084/m9.figshare.33122441).

Reviewer #1, Comment 2 — Survey weights

The authors state, "weighted estimates could be added if the editor prefers." While unweighted analysis is defensible given the within-population association aim, it would strengthen the manuscript to either conduct a sensitivity analysis using SHARE calibrated weights (if available), or more explicitly justify why unweighted analysis is appropriate.

Response. Addressed by adding the requested sensitivity analysis. We identified the SHARE Wave 9 calibrated cross-sectional individual weight (cciw_w9; calibrated to population totals by age, sex, and region) and re-fitted the primary confounder-adjusted model — which includes household wealth — using these weights, with standard errors clustered at the country level. The weighted estimate (OR = 0.78; 95% CI: 0.63–0.96; E-value 1.53 using the square-root OR-to-RR conversion, and 1.90 treating the OR directly as an RR) remained in the same direction and statistically significant, and was if anything slightly more protective than the unweighted primary estimate. It is now reported in S1 Table. We retain the unweighted model as primary, because the research question concerns a within-population association rather than nationally representative prevalence, and we now state this rationale more explicitly in the Methods and Limitations, noting that the weighted sensitivity analysis confirms the robustness of inference.

Reviewer #1, Comment 3 — Promote the flow diagram

Since the flow diagram is essential for understanding the sample derivation, it should be promoted to a main figure.

Response. Addressed. The participant flow diagram is now Figure 1, and all subsequent figures have been renumbered accordingly. All in-text references and the figure legends have been updated and cross-checked.

Reviewer #1, Comment 4 — DAG diagram

The paper's discussion acknowledges several plausible unmeasured confounders, yet these are not explicitly depicted in the DAG diagram. Adding them would enhance transparency. The DAG currently lists all mediators (BMI, smoking, etc.) as direct paths from the exposure to the outcome. However, in reality, these mediators influence each other (e.g., Depression → Physical Inactivity → BMI → Diabetes). Adding arrows between these mediators would make the causal structure more realistic.

Response. Addressed. We have revised the DAG (now S2 Fig) in two ways:

(a) We added an explicit node for unmeasured confounders (baseline frailty/functional reserve, personality traits, cognitive status, healthcare utilisation, and broader socioeconomic position), with arrows into both the exposure and the outcome, reflecting the plausible residual confounding discussed in the Limitations.

(b) We added inter-mediator arrows to represent the causal dependencies among mediators, including Depression → Physical inactivity → BMI → Diabetes (and Hypertension downstream of BMI/physical inactivity), while preserving acyclicity and retaining the exposure→mediator→outcome paths. The figure legend now describes these additions and clarifies that the mediator-adjusted models estimate a controlled direct effect and may be affected by overadjustment bias.

Reviewer #1, Comment 5 — S1 Table

Please confirm S1 Table is included and complete.

Response. Confirmed. S1 Table is included and complete. It reports, for the primary model: McFadden's pseudo-R² (0.06), the c-statistic (0.68), the Hosmer–Lemeshow goodness-of-fit result; per-variable missingness (all < 1.3%; highest for household wealth, 1.2%; 269/63,150 = 0.43% excluded for missing data); the primary caring×region cluster-robust Wald test; the country-level interaction sensitivity analysis, with the rank-deficiency caveat for the cluster-robust covariance matrix; the pairs-cluster and wild-cluster bootstrap, population-averaged (GEE), and random-intercept estimates; and the newly added weighted sensitivity estimate.

Reviewer #1, Comment 6 — Final consistency check of figure/table references

The authors should conduct a final systematic check of all figure and table references against the final PDF or Word document to ensure consistency.

Response. Addressed. We performed a systematic pass against the final PDF and confirmed that (i) every estimate reported in the text matches the corresponding table/figure, because all numbers derive from the single reproducible pipeline; (ii) all "Figure ??" placeholders have been resolved and figure numbering updated after promoting the flow diagram to Figure 1; (iii) table notes specify the exact covariate set of each model; and (iv) the terminology change to "any self-reported cardiovascular disease" is applied consistently across text, tables, and legends. We provide both a tracked-changes version and a clean final version.

Additional internal corrections (reported for transparency)

During the final consistency check we also resolved three further items:

• Author summary. The Author summary heading was previously empty; PLOS ONE requires this section for research articles, and we have now completed it (plain-language summary of why and how the study was done, the main finding, and its interpretation as hypothesis-generating).

• Financial disclosure. We aligned the submission-system Financial Disclosure with the cover letter. The statement now reads: "The authors received no specific funding for this work. EK is part-funded by the NIHR HealthTech Research Centre in Emergency and Acute Care (NIHR205301) and the Manchester British Heart Foundation Centre for Research Excellence (RE/24/130017). This work was also supported by a visiting research fellowship awarded by the University of Castilla-La Mancha."

• Geographic effect modification. During final verification, we identified that the previously reported caring×country interaction statistic was not appropriate for the clustered inference used throughout the study. With 28 country clusters, a full country-level interaction requires 27 additional parameters and yields a rank-deficient cluster-robust covariance matrix. We therefore assessed effect modification using a five-region caring×region interaction with a cluster-robust Wald test. This interaction did not reach conventional statistical significance (χ²(4) = 8.66, p = 0.070). The country-level analysis is retained in S1 Table for transparency, together with the note that its cluster-robust covariance matrix is rank-deficient and therefore unsuitable for primary inference. Alternative regional classifications likewise did not yield statistically significant interactions.

Response to Reviewer #2

Reviewer #2: The authors have responded thoroughly to the review comments, and I recommend acceptance of the revised manuscript.

We thank Reviewer #2 for the positive assessment and recommendation. No further points were raised; the present revision implements the Editor's and Reviewer #1's remaining requests while preserving the study's primary analytic framework and substantive conclusions.

We hope that these revisions satisfactorily address all remaining comments. We are grateful to the Editor and reviewers for their guidance, which has materially improved the manuscript, and we look forward to your decision.

Sincerely,

Raquel Cervigón & Evan Kontopantelis

Attachment Submitted filename: Response_to_Reviewers_R2.pdf

# Peer review: Decision Letter 2

**DOI:** 10.1371/journal.pone.0359074.r005

**Authors:** Mickael Essouma

10 Sep 2026

<p>Informal care outside the household by older people and cardiovascular health of caregivers: A Cross-Sectional Analysis of the SHARE European study

PONE-D-26-04680R2

Dear Dr. Cervigón,

We’re pleased to inform you that your manuscript has been judged scientifically suitable for publication and will be formally accepted for publication once it meets all outstanding technical requirements.

Within one week, you’ll receive an e-mail detailing the required amendments. When these have been addressed, you’ll receive a formal acceptance letter and your manuscript will be scheduled for publication.

An invoice will be generated when your article is formally accepted. Please note, if your institution has a publishing partnership with PLOS and your article meets the relevant criteria, all or part of your publication costs will be covered. Please make sure your user information is up-to-date by logging into Editorial Manager at Editorial Manager® and clicking the ‘Update My Information' link at the top of the page. For questions related to billing, please contact billing support.

If your institution or institutions have a press office, please notify them about your upcoming paper to help maximize its impact. If they’ll be preparing press materials, please inform our press team as soon as possible -- no later than 48 hours after receiving the formal acceptance. Your manuscript will remain under strict press embargo until 2 pm Eastern Time on the date of publication. For more information, please contact onepress@plos.org.

Kind regards,

Mickael Essouma, M. D.

Academic Editor

PLOS One

Additional Editor Comments (optional):

Reviewers' comments:

Reviewer's Responses to Questions

**Comments to the Author**

Reviewer #1: All comments have been addressed

**********

2. Is the manuscript technically sound, and do the data support the conclusions?

Reviewer #1: Yes

**********

3. Has the statistical analysis been performed appropriately and rigorously?

Reviewer #1: Yes

**********

4. Have the authors made all data underlying the findings in their manuscript fully available?

The PLOS Data policy

Reviewer #1: No

**********

5. Is the manuscript presented in an intelligible fashion and written in standard English?

Reviewer #1: Yes

**********

Reviewer #1: Journal: PLOS ONE

MS ID: PONE-D-26-04680R2

Title: Informal care outside the household by older people and cardiovascular health of caregivers: A Cross-Sectional Analysis of the SHARE European study

Version: R2 Date: 09 September 2026

Reviewer's report:

All my previous comments have been addressed.

**********

what does this mean?). If published, this will include your full peer review and any attached files.

If you choose “no”, your identity will remain anonymous but your review may still be made public.

**Do you want your identity to be public for this peer review?**  For information about this choice, including consent withdrawal, please see our Privacy Policy

Reviewer #1: **Yes:**  Mahmoud A. Abdel-Fattah

**********

# Editor report: Acceptance letter

**DOI:** 10.1371/journal.pone.0359074.r006

**Authors:** Mickael Essouma

PONE-D-26-04680R2

PLOS One

Dear Dr. Cervigón,

I'm pleased to inform you that your manuscript has been deemed suitable for publication in PLOS One. Congratulations! Your manuscript is now being handed over to our production team.

At this stage, our production department will prepare your paper for publication. This includes ensuring the following:

* All references, tables, and figures are properly cited

* All relevant supporting information is included in the manuscript submission,

* There are no issues that prevent the paper from being properly typeset

You will receive further instructions from the production team, including instructions on how to review your proof when it is ready. Please keep in mind that we are working through a large volume of accepted articles, so please give us a few days to review your paper and let you know the next and final steps.

Lastly, if your institution or institutions have a press office, please let them know about your upcoming paper now to help maximize its impact. If they'll be preparing press materials, please inform our press team within the next 48 hours. Your manuscript will remain under strict press embargo until 2 pm Eastern Time on the date of publication. For more information, please contact onepress@plos.org.

You will receive an invoice from PLOS for your publication fee after your manuscript has reached the completed accept phase. If you receive an email requesting payment before acceptance or for any other service, this may be a phishing scheme. Learn how to identify phishing emails and protect your accounts at https://explore.plos.org/phishing.

If we can help with anything else, please email us at customercare@plos.org.

Thank you for submitting your work to PLOS One and supporting open access.

Kind regards,

PLOS One Editorial Office Staff

on behalf of

Dr. Mickael Essouma

Academic Editor

PLOS One
